# Local microglial activation induced and labeled in the retina in a novel subretinal hemorrhage mouse model

**DOI:** 10.1038/s41598-025-09007-w

**Published:** 2025-07-10

**Authors:** Boglárka Balogh, Marietta Zille, Gergely Szarka, Loretta Péntek, Anett Futácsi, Béla Völgyi, Tamás Kovács-Öller

**Affiliations:** 1 Szentágothai Research Centre, University of Pécs, Pécs, Hungary; 2 Department of Neurobiology, Institute of Biology, Faculty of Sciences, University of Pécs, Pécs, Hungary; 3 Division of Pharmacology and Toxicology, Department of Pharmaceutical Sciences, University of Vienna, Vienna, Austria; 4 NEURON-066 Rethealthsi Research Group, Pécs, Hungary; 5 Imaging Core Facility, Szentágothai Research Centre, University of Pécs, Pécs, Hungary; 6 Medical School, University of Pécs, Pécs, Hungary

**Keywords:** Bleeding, Blood, Cholera toxin, Experimental ophthalmology, Eye, Inflammation, Retinal pigment epithelium, Neuroimmunology, Visual system, Cell biology

## Abstract

Subretinal hemorrhage (SRH) is caused by the accumulation of blood between the neurosensory retina and the retinal pigment epithelium or between the retinal pigment epithelium and the choroid. SRH often arises from age-related macular degeneration, traumas, and may occur spontaneously caused by other diseases like hypertension and diabetes. Here, we developed a novel technique - co-injection of blood and a dye-coupled tracer protein, Cholera toxin subunit B (CtB) - to better localize and understand the disease and how it can cause microglial activation, inflammation, and partial vision loss. Our results show that microglia are activated in the inner retinal layers in zones adjacent to blood injection. In contrast, the non-affected zone of the injected eye showed no microglial activation. For the first time, we used phosphate-buffered saline (PBS) injections as a control to assess the specific effects of injected blood. The results demonstrated that blood induced a markedly stronger activation response in the surrounding tissue, whereas PBS elicited a comparatively milder effect. PBS did cause microglial activation, but it was largely confined to the injection site and adjacent regions, and to a lesser extent than that observed with blood. We also observed microglial activation in the inner retina, along with the emergence of microglia and macrophages in the retinal pigment epithelium. Using advanced imaging techniques, we were able to better localize the affected area which comprises not only the immediate retinal area over the blood clot but the neighboring regions as well. These findings will provide the basis for novel therapeutic interventions targeting neuroinflammation in the retina after subretinal hemorrhage and other diseases affecting the eye.

**Supplementary Information:**

The online version contains supplementary material available at 10.1038/s41598-025-09007-w.

## Background

The retina is a gateway to all visual information. It is, however, one of the most exposed parts of the central nervous system (CNS)^1^, and thus susceptible to diverse retinal diseases and environmental insults^2^. Bleeding in the eye can arise from various etiologies, including trauma, systemic diseases, and ocular disorders, and in most cases, leads to major vision loss or blindness^3–6^. Retinal hemorrhages can be classified based on their location within the retinal layers: preretinal, intraretinal, and subretinal.

**Fig. 1:**
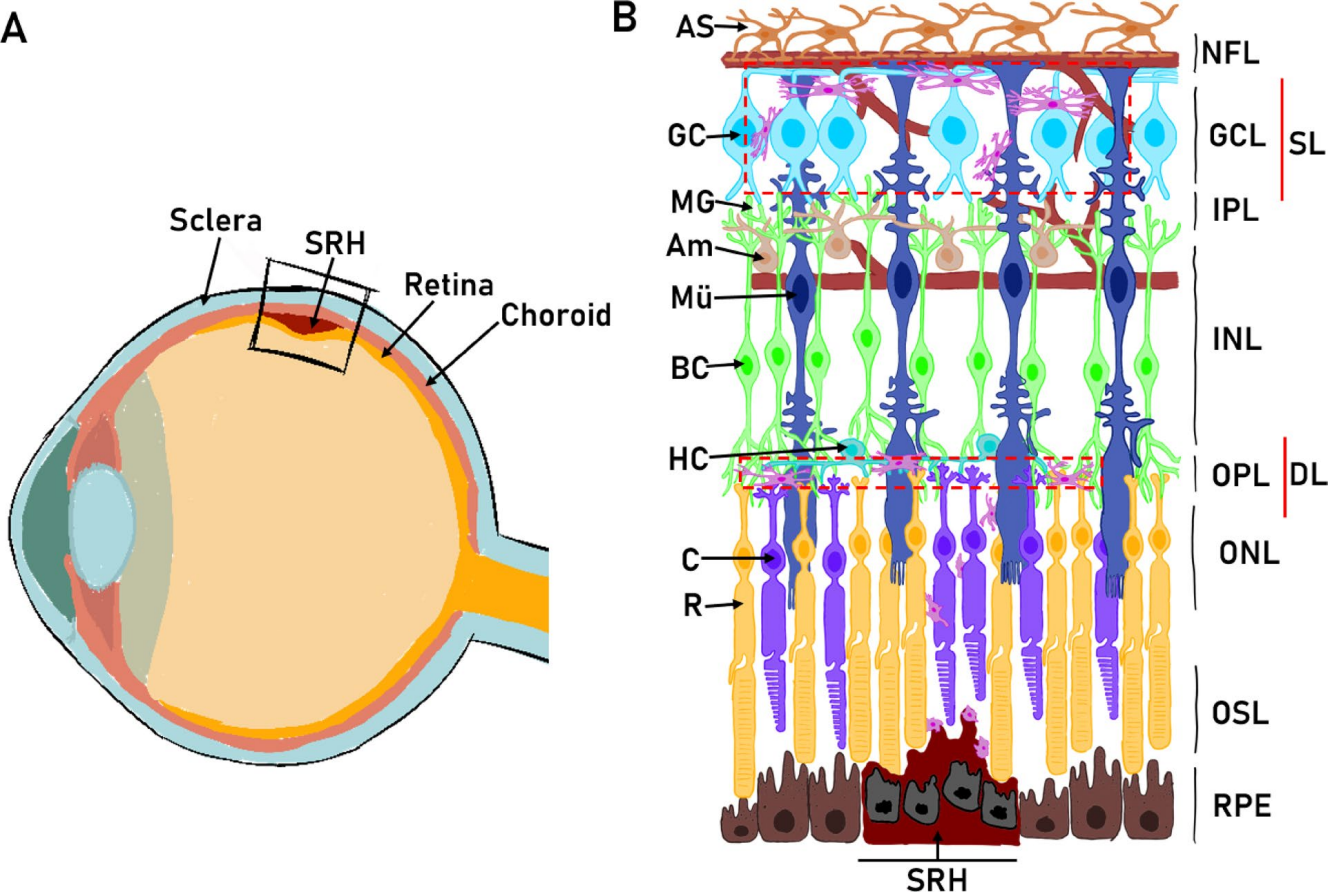
Subretinal hemorrhage. (**A**) Schematic diagram represents the human eye and (**B**) the retinal layers with the respective cell types. Dashed red boxes show microglia layers in the healthy retina. Abbr. Astrocyte (AS), Ganglion Cell (GC), Microglia (MG), Amacrine Cell (Am), Bipolar Cell (BC), Horizontal Cell (HC), Cone (C), Rod (R). Neurofilament Layer (NFL), Ganglion Cell Layer (GCL), Inner Plexiform Layer (IPL), Inner Nuclear Layer (INL), Outer Plexiform Layer (OPL), Outer Nuclear Layer (ONL), Outer Segment Layer (OSL), Retinal Pigment Epithelium (RPE), Subretinal Hemorrhage (SRH). Dashed red lines indicate the Superficial Layer (SL) and Deep Layer (DL) of microglia.

Subretinal hemorrhage (SRH) is the accumulation of blood between the retina and the retinal pigment epithelium (RPE) or between the RPE and the choroid^7^ (Fig. 1). The presence of blood in this space is toxic to retinal cells, and rapid tissue damage is induced starting with photoreceptor and RPE degradation^8^. If the clot is left untreated, scarring processes are initiated potentially leading to visual loss in the affected area. If the hemorrhage occurs in the macular region, it usually leads to severe and irreversible vision loss^9^. However, the visual prognosis in all cases depends on the initial visual acuity, the size of the hemorrhage, the time interval between the onset of SRH and treatment, and the concomitant involvement of different retinal segments. The development of SRH may be associated with several pathologies such as age-related macular degeneration, hypertension, diabetic retinopathy, or even blunt head trauma^10^.

The retinal tissue damage is facilitated by three main pathological pathways: (1) The clot acts as a diffusion barrier, which prevents the transfer of nutrients from the choroid to the photoreceptors by the RPE and the return of by-products to the choroid^10,11^; (2) Mechanical effects are caused by the contraction of the fibrin network formed after the clot, which can tear the photoreceptor outer segment from the retina in sheets^10^; (3) Lysis of erythrocytes results in the release of hemoglobin, which is no longer protected, and the iron in the heme is oxidized from the Fe^2+^ state to the Fe^3+^ state^12^. Oxidized heme and other blood breakdown products have been demonstrated to induce cell death of the RPE and photoreceptors^8^. In addition, other cellular changes are observed, including activation and infiltration of microglia and macrophages, Müller cell disposition, presence and activation of migrating RPE cells, suggestive of dedifferentiation and signs of gliosis^13^. As a result, nerve cells will suffer from severe inflammation and cell death.

Microglia are the resident immune cells in the central nervous system, including the retina. In the human retina, they form a heterogeneous population; perivascular microglia filter substances that enter the retina through the vasculature, whereas parenchymal microglia have high motility, constantly monitoring the microenvironment to remove possible metabolic products and cellular debris and mediate synaptic remodeling^14^. Microglia are also programmed for immunological tolerance and possess an anti-inflammatory phenotype, which is essential for retinal immune competence^15^.

Several factors are involved in the crosstalk between retinal microglia and their environment. CX3CR1 plays a role in the pathophysiology of cerebral ischemia^16^. Müller cell activation in mice induced microglia activation through the ATP/P2X7 receptor pathway, while the release of cytokines such as tumor necrosis factor and interleukin-6 can exacerbate retinal damage^17^. Microglia are involved in pruning weak presynaptic terminals of retinal ganglion cells^18^. CD200 (or OX2) can prohibit microglia from tissue damaging activation through negative feedback^19^.

Because of their monocytic origin, microglia can respond incredibly quickly to pathological stimuli, therefore, they are excellent indicators of retinal pathologies, including inflammation. Microglia protect neurons from cell death and promote tissue regeneration but can also induce secondary tissue damage by triggering chronic inflammation^20–23^. They can take different morphological states; each morphotype is associated with different activation states and has unique functions to maintain a physiologically normal state^24–27^.

In this study, we utilized microglia as indicators to assess retinal activation induced by SRH, based on their morphotypes and movement in the tissue. To enable this analysis, we developed a novel co-injection model, as existing SRH models did not permit direct fluorescent labeling of the injection site, not to mention microglial activation through labeling with phagocytosis of the dye-toxin-subunit complex.

## Materials and methods

### Ethics statement

Animal housing, handling, and all utilized experimental procedures were approved by the ethical committee of the University of Pécs (BA02/2000-27/2024), and in accordance with the ARRIVE guidelines and regulations^28^. All procedures conducted in accordance with the ARVO Statement for the ‘Use of Animals in Ophthalmic and Vision Research guidelines and regulations. All efforts were made to minimize pain and discomfort during the experiments and all procedures were done by obeying the 3R law. No experiments were done involving human participants.

### Animals and Preparation

Adult, male, P30-90 old C57BL/6J (*n* = 10) mice, bred in the Animal Core Facility of the Szentagothai Research Centre of the University of Pécs from inbred parents originally acquired from Jackson Laboratory (JAX, Bar Harbor, ME, USA) were used. Mice were maintained in a 12/12-hours dark/light cycle. Max. 4 mice were placed in a cage with food and water *ad libitum*.

**Fig. 2:**
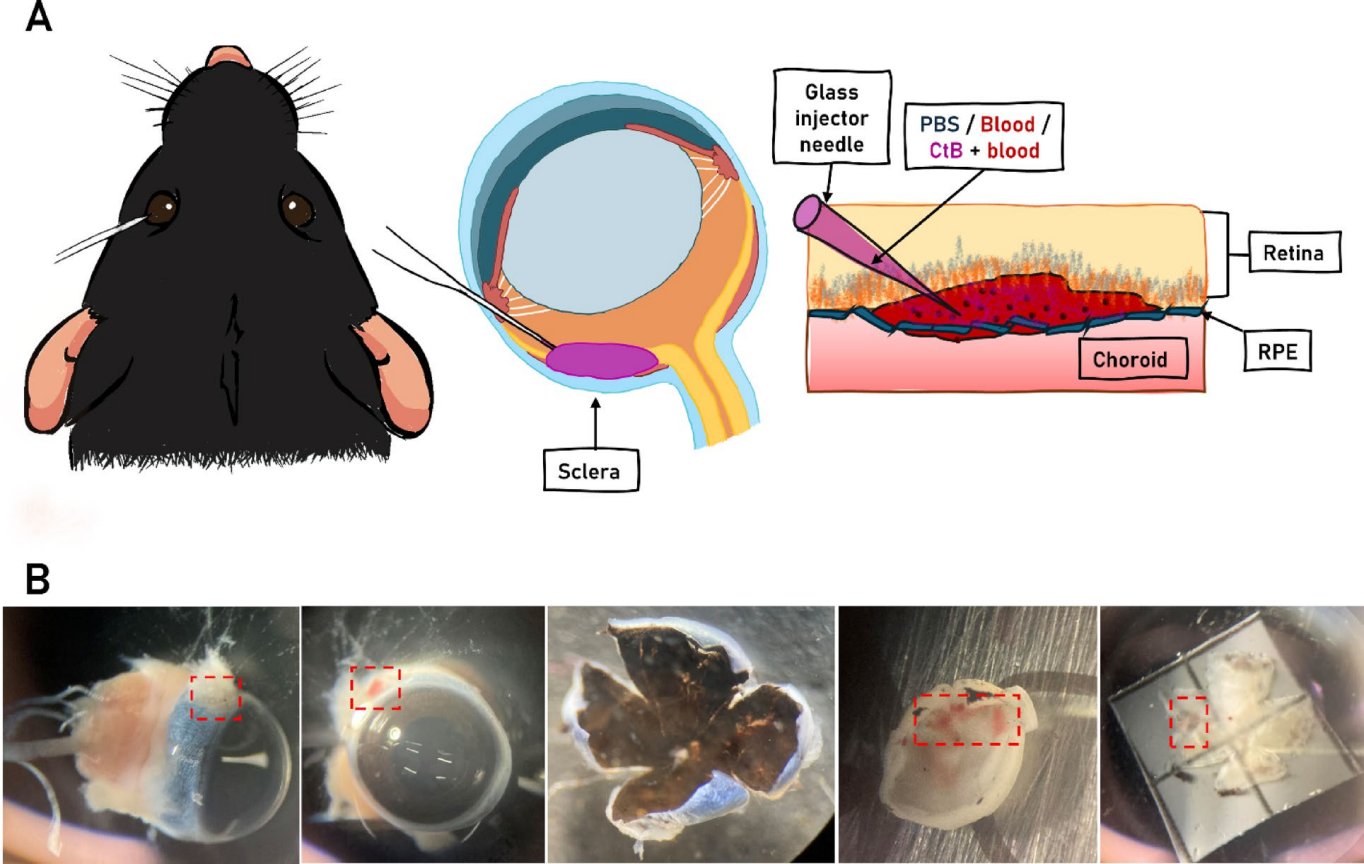
Subretinal injection in the mouse. (**A**) SRH injections. (**B**) Stereo-microscopic images during the eye dissection validating the injection site and the presence of subretinal blood (*). Dashed red areas show the SRH sites, demonstrating the healing of the injury at the ora serrata.

To create the mouse model of SRH, animals were deeply anesthetized via intraperitoneal injection of pentobarbital (80x dilution from stock (400 mg/ml) → for mice 180 µl/10 g). For positive controls, autologous blood was collected from the tail vein with a 27-gauge needle and immediately used for subretinal injections. Before the subretinal injections, a temporal tunnel incision posterior to the limbus was made in the sclera using a 27-gauge needle under a dissecting microscope. The subretinal injections of autologous blood were achieved with a glass injector needle connected to a 1 ml syringe. Glass injector needles were pulled with a Sutter P-87 (program: Cycle1: Heat = 760, Pull = 250, Vel.=50, Time = 200; Cycle2: Heat = 760, Pull = 250, Vel.=0, Time = 200) from 1.5 mm capillaries (World Precision Instruments, Inc. Item Nr. 1B150F-4, Lot Nr. 2605323). For the control SRH group, we injected 2 µl blood into the subretinal space. During the co-injection experiments, we loaded the glass injector with 0.25 µl of CtB conjugated with Alexa-555 solution (CtB-A555, cc.: 1 µg/µl in PBS, Invitrogen, Lot Nr. 201622) followed by 2 µl of blood (Fig. 2A). Negative controls (SHAM) injections were done by injecting 2 µl of PBS subretinally. To ensure that no blood effect was recorded in our controls, we used albumin labeling to check for any extravasal blood in the PBS injections. We excluded all samples where we found albumin positivity in the tissue, except for the inner retinal blood vessels. We performed the SRH and PBS injections only on the left eyes, while the right eyes were used as internal null-controls.

We created 4 groups. Group 1 was subretinally injected with the Blood + CtB-A555 mixture, as described (*n* = 5, 2 unit. 2 + 3 mice), Group 2 was injected with blood only (*n* = 3, 1 unit), Group 3 was the non-injected control (*n* = 2, 1 unit), and Group 4 was the negative control (SHAM, PBS) injections (*n* = 5, 1 unit). All eyes (26 pcs) were used and as a result we had 5 experimental settings: (1) Blood + CtB-A555 (*n* = 5), (2) Blood only (*n* = 3), (3) Internal controls from treated mice (*n* = 8), (4) Non-treated (*n* = 4), (5) SHAM treated (*n* = 5). We also jointly tested 2 pigmented epitheliums from the 1. setting.

To avoid secondary inflammatory effects and only focus on microglial activation, we chose a 24-hour survival time based on the literature^29,30^. Mice were sedated with Forane (isoflurane 4%, 200 µl/l) and then sacrificed using cervical dislocation. Eyes were immediately removed with fine forceps. Eyeballs were cut at the ora serrata, and the lens and vitreous body were removed. The retinas and the attached pigment epithelium sheets were fixed in 4% paraformaldehyde on filter papers for 30 min at room temperature (RT) (Fig. 2B).

### Immunohistochemistry

Flat-mounted retinas and RPE were blocked in blocking solution (CTA, containing 5% Chemiblocker, 0.5% TritonX-100, 0.05% Na-azide in PBS) for 2 h at room temperature, humidified. After blocking, the retinas and RPEs were treated with the primary antibodies (Table 1; diluted in CTA) for 48 h at RT. After washing three times in PBS, secondary antibodies (Table 1) were applied in CTA, and incubated overnight at RT. After washing three times for 10 min in PBS, they were coverslipped with the ganglion cell layer (GCL) facing up, or RPE up with Vectashield (Vector Laboratories, Peterborough, UK).

**Table 1:** Antibodies.

Primary Antibodies					
Name	Abbr.	Dilution	Source	Code	RRID
Ionized calcium-binding adapter molecule 1	gp-Iba1	2000x	SySy	234,004	AB_2924932
Junctional adhesion molecule B	gt-JAM-B	1000x	R&D sys	AF988	AB_355767

**Secondary Antibodies**					
**Name**	**Abbr.**	**Dilution**	**Source**	**Code**	
Anti-guinea pig - Alexa647	a-gp-A647	500x	Invitrogen	A21450	
Anti-mouse - Alexa405	a-ms-A405	500x	AbCam	ab175658	
Anti-goat - Alexa647	a-gt-A647	500x	AbCam	AB150131	
Anti-guinea pig - DyLight405	a-gp-DL405	500x	Jackson	706-475-148	

### Microscopy

Retinas and RPEs were inspected using a Zeiss LSM 710 confocal laser scanning microscope (with Plan Apochromat 10x, 20x, and 63x objectives (NA: 0.45, 0.8, 1.4, Carl Zeiss Inc., Jena, Germany)) with normalized laser power and filter settings making 1.5- and 0.5-µm thin optical sections.

### Image analysis

All measurements were performed using FIJI (ImageJ 1.54, NIH, Bethesda, MD, USA). First, we performed two z-merges from consecutive optical stacks for the superficial and deep regions of microglia from 3 areas from the treated eyes (Z1-3, starting with B when blood was injected and P when PBS as SHAM control) and one area from the control eyes (Z4). We also overlapped the injection site (Z1) with blood albumin (a-albumin staining). The size was variable, depending on the injection. Z2 was determined as an area (700 × 700 μm) in connection with the injection site but with no extravasal albumin present. Z3 was the distal-most relative to the injection site (contralateral retinal hemisphere) in the sample (700 × 700 μm).

We used the 20x magnification for clustering microglia activation. Microglia were divided into superficial (SL) and deep layers (DL) defining their positions in the retina, represented by the z-stack regions from GCL, inner plexiform layer, neuronal layers, and the superficial layer of the inner retinal vasculature when merging images for SL-microglia and the outer plexiform layer/DL neuronal layers together with the deep layer of inner retinal vasculature to merge the microglia images for the DL from the same Z-stack. Microglia were separated from the other signals based on their expression of ionized calcium-binding adapter molecule 1 (Iba1). Cells were manually grouped one by one according to their morphologies into activated and non-activated see the details of cell classification in^31^, using the “Cell-counter” plugin in FIJI. Only fully visible cells were included in the analyses, while we omitted those that fell on the edges. Additionally, as a form of further validation to show that the classification of cells was objective and gives the same outcome without human interference, we used a MG morphometric and motility toolset plugin: MotiQ^32^ to analyze our sample set of images, where 15–15 cells were randomly selected from all groups and subjected to morphometric analysis. Supplementary Fig. 2.

Motion was detected on 15 s. time-lapse images. We decoded the change in Z with ImageJ ΔF/F script (by^33^).

#### Statistics

Sample sizes were determined based on previous power analysis (G*Power, HHU, Düsseldorf, Germany). Data was curated in MS Excel (Office365, Microsoft, Redmond, WA). Non-parametric Kruskal-Wallis and Dunn’s posthoc analyses were performed using IBM SPSS Statistics Version 25.0 (IBM Corp., Armonk NY) and JASP Version 0.19.3 (JASP Team, 2024). ANOVA and Tukey’s post hoc analyses were performed using JASP Version 0.19.3. (JASP Team, 2024). Normality tests were performed before the analyses, because some of the data sets showed non-normal distribution, we chose to perform non-parametric tests on all data. We were not able to use randomization due to pathological and staining differences in the retina of different groups.

## Results

### A novel SRH model

Based on previous studies (see details in Table 2), we have developed a modified model for SRH, where compared to previous methods, we used freshly pulled glass injectors to avoid complications after injection and labeled the injection site with the co-injection of CtB-A555 toxin subunit conjugate to allow better visualization of the injection site and the phagocytosis of the dye in the live retina.

**Table 2:** Previous SRH models.

Species (references)	Method	Results	Limitations
**Mouse** ^34^	Injections via transscleral route with a 33G needle 1.6 µl autologous blood (from tail vein); survival times 6 h to 10 days	Increased expression of inflammatory cytokines, chemokines and adhesion molecules; microglial migration, increased density; effects of minocycline	Use of metal needle, lower biocompatibility and larger tip size
**Rat** ^35^^–^^37^	heparinized and diluted blood (one part blood to ten parts saline) injected subretinally	The retina overlying the hemorrhage became intensely degenerated over a period of months	Heparin is an anticoagulant; clotting process differs from fresh blood injections
**Rabbit** ^38^	0.1 ml fresh blood from ear; specially constructed 30G needle tip encased in a 22G spinal needle; clot prevention 0.03 cc of air in the needle tip (also injected in the subretinal space); survival times:1 h to 28 days	Signs of degeneration after 1 day in the outer retinal layer	Use of plus air bubble, can make retinal detachment worse
**Rabbit** ^39^	0.1 ml fresh blood from ear; 30G needle; Clot prevention: air bubble	Intravitreal injection of tPA 1 day after subretinal injection of blood in rabbits facilitated more rapid lysis of the clotted blood, however, retinal damage was not prevented	Use of plus air bubble, can make retinal detachment worse
**Rabbit** ^40^	100 µl autologous blood from ear vein in a nasal juxtapapillary location along the myelinated streak; An angled 1-inch, 30-gauge needle was introduced transsclerally 3–4 mm posterior to the limbus	Photoreceptor toxicity caused by SRH occurs at least in part by apoptosis and is associated with iron migration to the photoreceptor layer	Use of metal needle
**Cat** ^41^	the tip of a 20-gauge surgical knife or a 25-gauge needle was passed transsclerally into the bleb and then withdrawn, allowing choroidal blood to extend under the retina into the area centralis; Lesions were observed 25 min to 14 days	In 6 of 9 clots more than 1 h old, fibrin was associated with tearing of sheets of photoreceptor inner and outer segments. Later degeneration progressed to involve all retinal layers overlying the densest areas of fibrin in the clots; hemorrhages into subretinal blebs containing tPA did not form fibrin strands or cause photoreceptor tearing	Use of metal needle
**Cat** ^42^	the tapetal or nasal retina with a neodymium: YAG (Nd: YAG) laser focused through a preformed retinal bleb; 2–25 laser shots (mean, 14) at between 20 and 25 mJ were fired until one created a large subretinal hemorrhage	Removing experimental SRHs within 7 days of their occurrence with the assistance of rt-PA and an ultra-microsurgical approach may reduce outer retinal degeneration	Laser due to heat and tissue absorption

Injection sites of all injected mice healed entirely without any complications or infection. The entry site on the eyeballs were closed within 24 h with only mild visible post-operational scar that could be discerned only under stereo microscope after careful examination. The presence of the scar also served to localize and validate the injection site and the presence of subretinal blood in the eyecup before fixing with 4% paraformaldehyde or removing the retina for live imaging (Fig. 2).

**Fig. 3:**
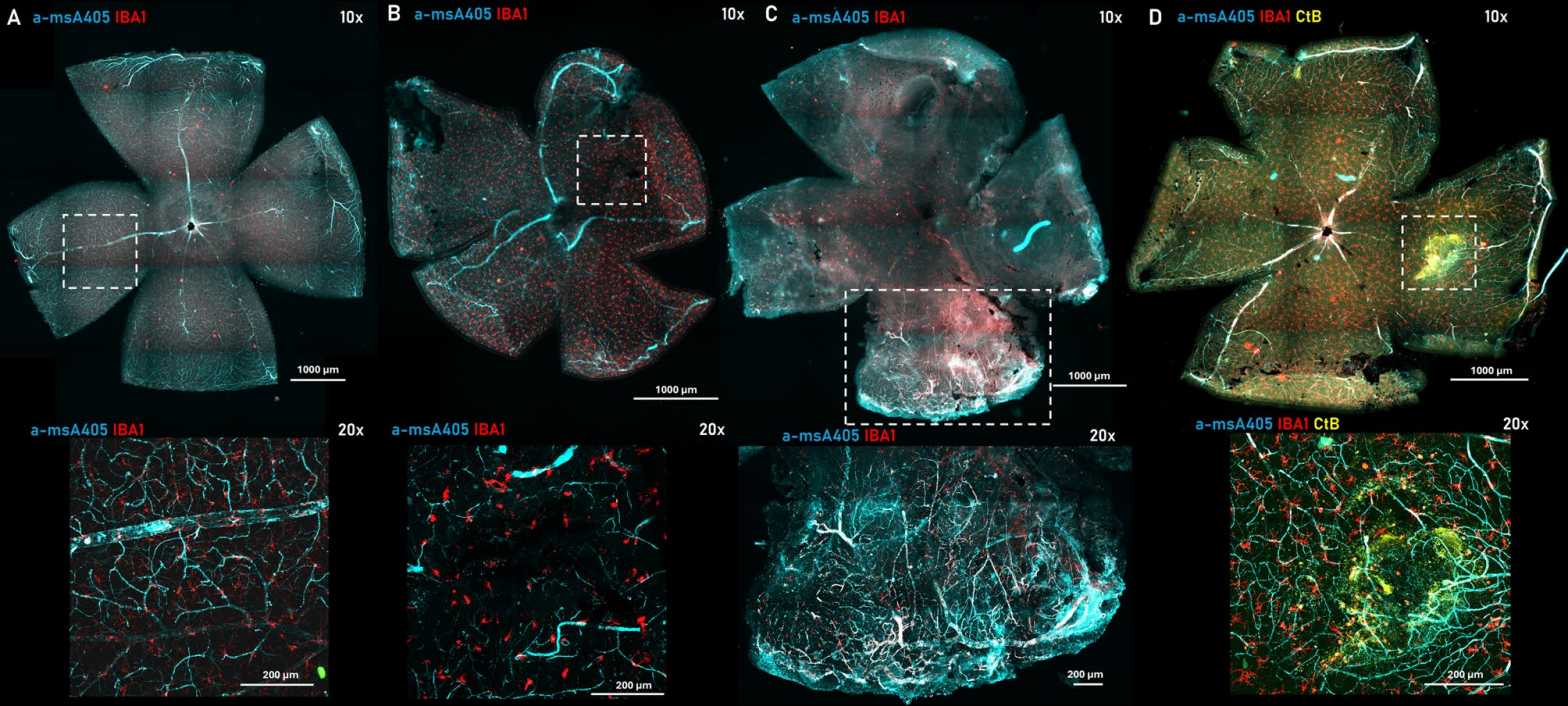
Control, SHAM (PBS), SRH - blood, SRH - CtB injection sites. Whole mount control (**A**), SHAM (PBS) injected (**B**), blood injected (**C**) and blood/CtB-A555 co-injected (**D**) retina. The dashed white square (top row) shows the injection site, while the image below (bottom row) focuses on the injection site in 20x magnification of the same sample.

To our knowledge, this is the first study to use PBS injection as a positive control (sham) to distinguish blood-induced effects from those caused by the physical stress of the injection procedure. PBS injection did not elicit clear signs of secondary microglial activation; observable activation was limited to the glass needle’s exit site (Fig. 3B).

**Fig. 4:**
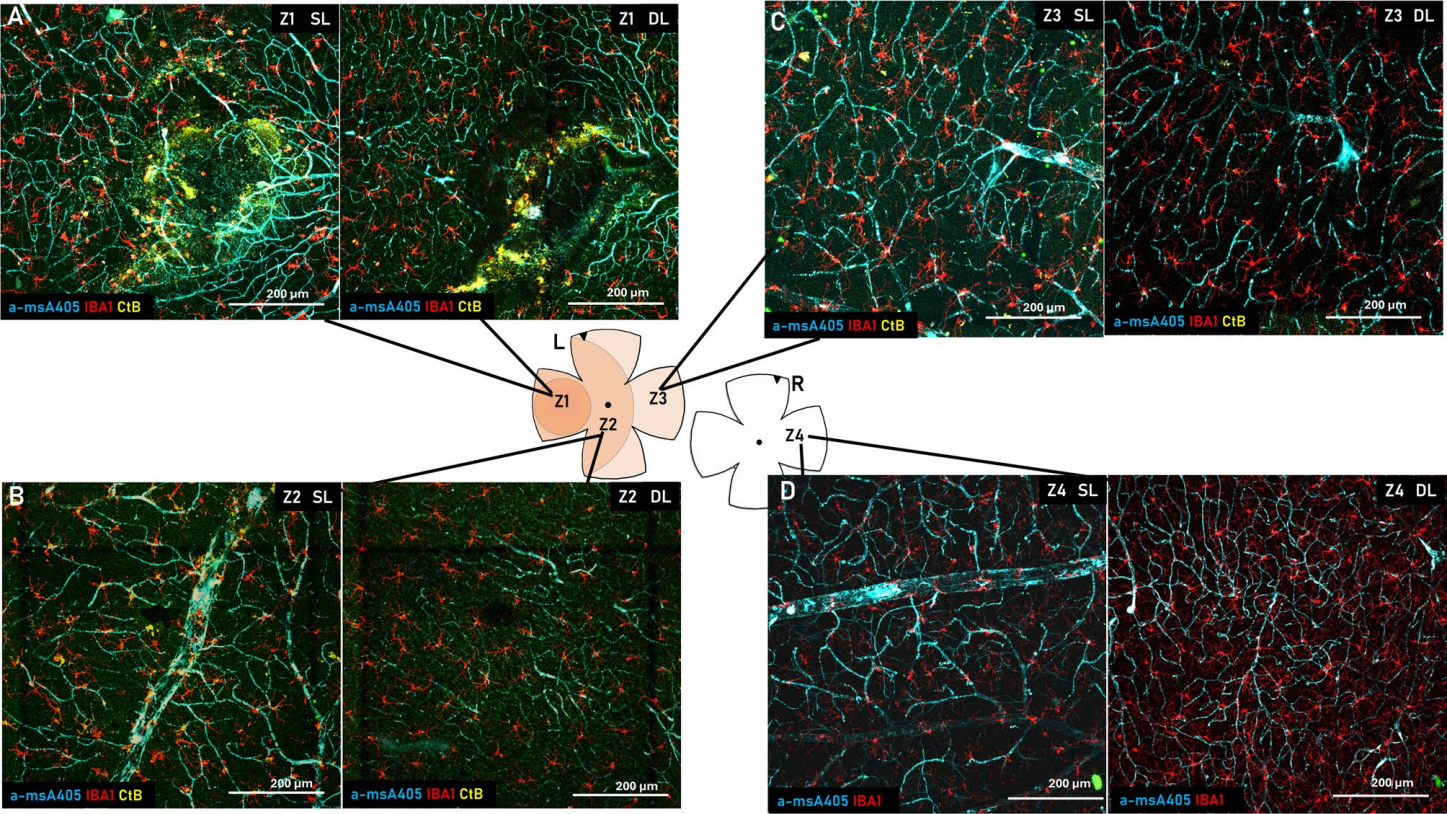
Example microscopic images of the SRH site and the surrounding areas. (**A**) SRH site (Z1) at the level of the superficial layer (left) and the deep layer (right). (**B**) Neighboring zone (Z2) superficial layer (left) and the deep layer (right). (**C**) Z3, the farthest zone from the SRH site at the level of the superficial layer (left) and the deep layer (right). (**D**) Control retina (Z4; contralateral eye) at the level of the superficial layer (left) and the deep layer (right). All images are 20x magnification confocal images.

As another novelty, we used CtB for the first time to label the injection site in the subretinal space and the overlying cells, therefore, the retinal localization of the injection can be precisely outlined. Using this approach, we have defined three zones in the injected eye, including the injection site (Z1), the neighboring site, in direct contact with the injection site (Z2), and the corresponding contralateral region within the same retina (Z3). We added an endogenous control zone from the contralateral, non-injected eye (Z4) (Fig. 4).

We observed that the CtB-A555 not only overlapped with the blood-related extravasal albumin (Fig. 3) but also provided a superior Z1 vs. Z2 contrast to the albumin signal based on laser intensity and gain settings. Consequently, the SRH site was more readily demarcated than in non-co-injected retina, where only intravasal albumin was present (Fig. 3A). We did not identify any blood-related extravasal albumin deposits in the contralateral side (Z4) retinas, used as an endogenous negative control (Fig. 3A). Both blood injection and Blood + CtB injection had apparent signs of injury and related microglial activation (Fig. 3C, D) compared to the PBS control (Fig. 3B).

By using higher magnification (20x) each area can be easily delineated with the CtB-A555, enabling reliable classification of individual microglia in the four zones (Z1-4). Accordingly, our measurements accurately represent the microglial states in each of the zones (Fig. 4). As the example images show the microglial cells from the SL, which lie on the NFL side of the GCL, exhibit pronounced activation. This activation is also evident in the deep layer (DL) at the interface between the inner nuclear layer and the outer plexiform layer (Fig. 4a). The two layers of microglia shown in the healthy retina broke up and became disordered in Z1 while in Z2 the SL-DL division was still intact with more activated microglia present (Fig. 4b) compared to both Z3 and Z4.

### Retinal microglial activation after SRH

**Fig. 5:**
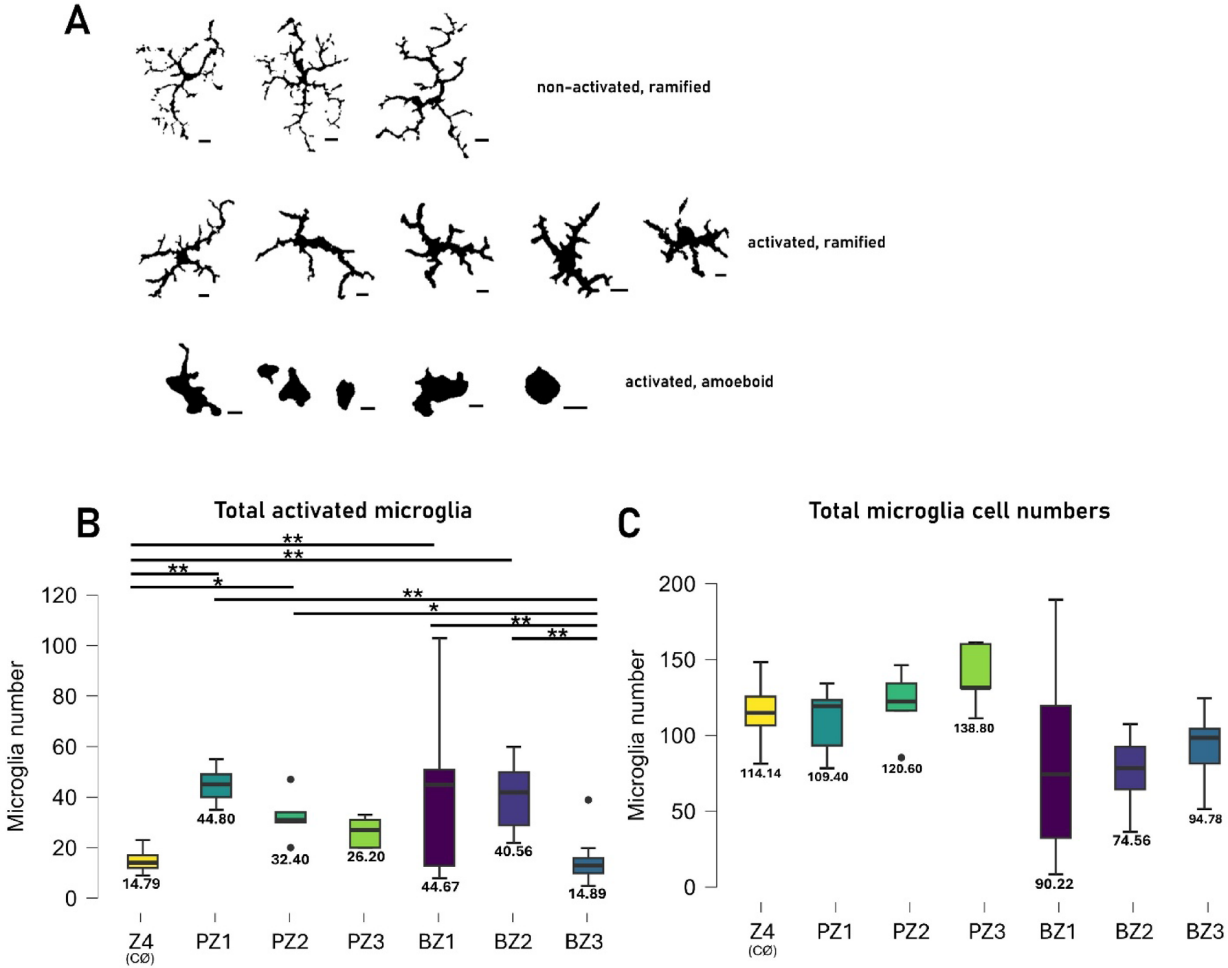
Change in the number of activated microglia due to SRH. (**A**) Typical morphological changes from our dataset, ranging from non-activated (top row), ramified (middle row) to activated, amoeboid (bottom) scale bar: 10 μm. (**B**) The SRH (BZ1) site showed a pronounced increase in the activated microglia count in comparison to all other SRH zones, including neighboring (BZ2), off-site (BZ3), and contralateral retinal (Z4). PBS injected and neighboring retinal zones (PZ1-2) showed a significant, but mild increase in activated numbers versus Z4. (**C**) After 24 h, we detected a small decrease in the total number of microglia in BZ1-4. Interestingly, a small, non-significant decrease was detectable in the PBS injected eye (PZ1-3) vs. the contralateral eye (Z4) in the total number of microglia. Kruskal-Wallis, Dunn’s post hoc **p < 0.01 o = outlier x = mean. Original dataset available in the Supplement.

Based on morphological classification (previously described in^31^) of microglia (Fig. 5A), SRH induced microglial activation in BZ1 and BZ2 was present in both the SL and DL (Fig. 5B). We observed a 3.02-fold increase in the number of activated microglia in BZ1 (from a mean of 14.79 to 44.67) as well as a 2.74-fold increase in BZ2 (from 14.79 to 40.56) when compared to the control Z4 sample of the contralateral eyes. In addition, we detected an increase in the number of microglia between the BZ1 and the BZ3 and between the BZ2 and BZ3 (Z3-Z1: 3.00-fold, from 14.89 to 44.67; Z3-Z2: 2.72-fold, from 12.89 to 40.56). A similar comparison of BZ3 and Z4 revealed no significant difference in the number of the activated microglia, indicating that microglia in these regions lack activating signals and retain non-activated morphologies. While total microglial counts did not differ significantly across the zones, we observed an increased variability of the total microglia counts within BZ1 across retinas (Fig. 5C, *n* = 5778 cells in 28 retinas)^32^.

PBS did not cause high variance in PZ1 and the PZ2 activation was less pronounced. Cell numbers were somewhat less in BZ1-3 but without statistical significance.

**Fig. 6:**
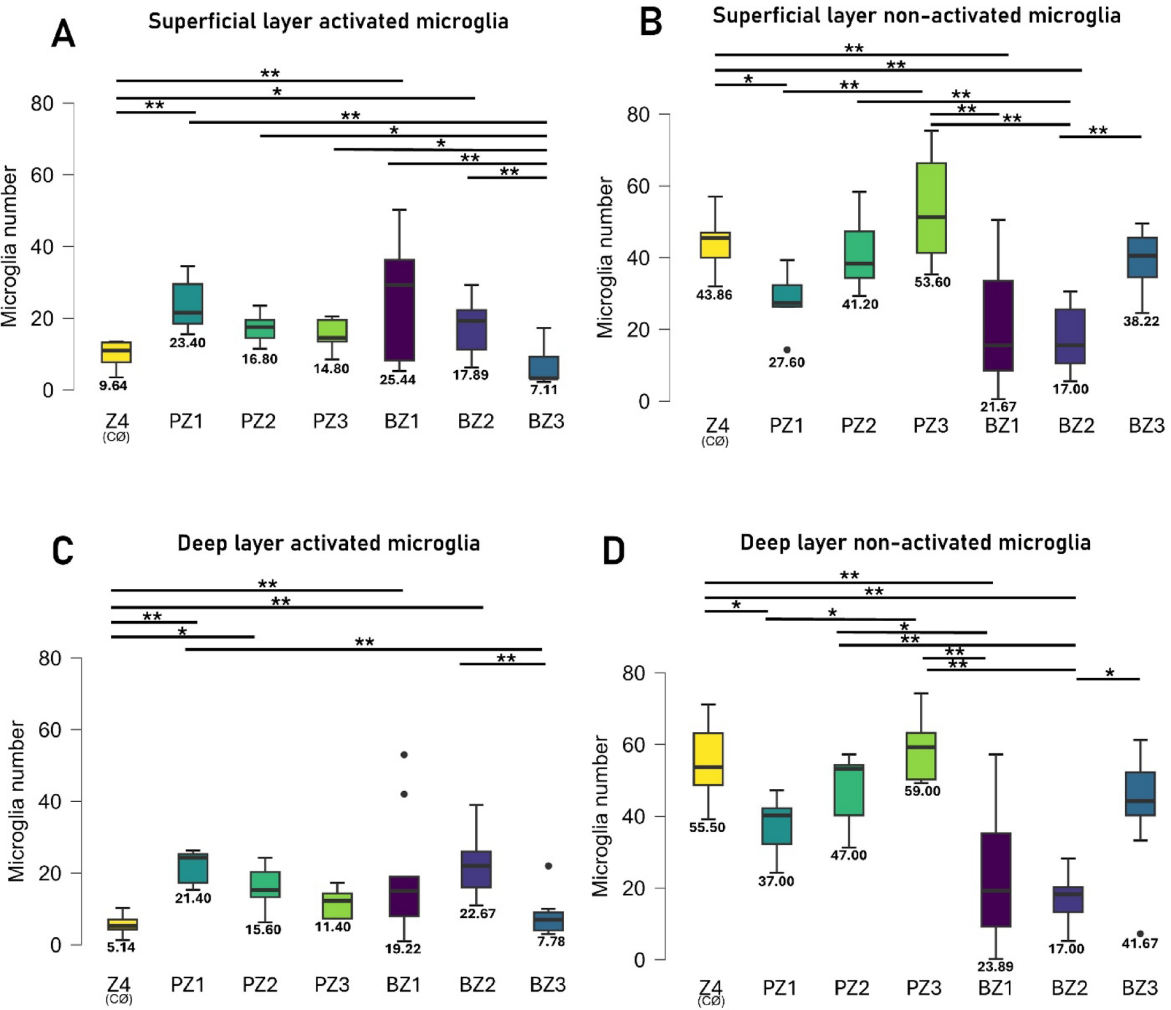
Change of activated microglia counts in the superficial (SL) and deep layers (DL) of the SRH inner retina.** A-B**) SL microglia (MG) activation states in different zones.** C-D**) DL microglia distribution in the different zones and activation states. BZ indicates SRH-blood injection, PZ indicates PBS SHAM-injection. Z4 = non-treated contralateral retina. For zone details see Figs. 4 and 5. Kruskal-Wallis, Dunn’s post hoc **p < 0.01 *p < 0.05 o = outliers x = mean. Original data available in the Supplementary Dataset.

Interestingly, when we examined the microglial activation in the SL and DL, we observed a pronounced change between Z4 (non-treated Ctrl) and BZ1 (blood injected zone). We only detected a significant difference between BZ1 and BZ3 in the number of activated microglia in the SL, BZ1-3 activation profile looked similar to PZ1-3 with slightly more numerous activated cells when injected with blood at the injection site (PZ1 vs. BZ1: 1.09-fold increase, from 23.40 to 25.44) and next to it (PZ2 vs. BZ2, 1.06-fold, 16.0 vs. 17.89 (Fig. 6A**)**). On the other hand, the counts of the non-activated microglia in the SL were significantly lower in BZ1-2 when compared to either PZ3 or Z4 (Fig. 6B, BZ1-PZ3: 2.47-fold 21.67 vs. 53.60, BZ2-PZ3: 3.15-fold, 17.00 vs. 53.60, BZ1-Z4: 2.02-fold, 21.67 vs. 43.86, BZ2-Z4: 2.58-fold, 17.00 vs. 43.86). In summary, the net activation of microglia in the SL shows a similar pattern to the PBS-SHAM (PZ1-3) with slightly increased numbers in SRH.

When examining the DL, the number of activated microglia significantly increased, in contrast to Z4 at the injection sites (BZ1-Z4: 3.74-fold, from 5.14 to 19.22 & PZ1-Z4: 4.16-fold, from 5.14 to 21.40) and next to it (BZ2-Z4: 4.41-fold, from 5.14 to 22.67 vs. PZ2-Z4: 3.04-fold, from 5.14 to 22.67) (Fig. 6C). Similarly to the SL, the number of non-activated microglia of BZ1 and BZ2 counts significantly decreased compared to Z4 (Fig. 6D, Z4 - BZ1: 2.32-fold, from 55.50 to 23.89; Z4-BZ2: 3.26-fold, from 55.50 to 17.00). The total microglial numbers decreased at the injection sites with an average of 1.27-fold at BZ1 and 1.04-fold at PZ1. The numbers also decreased at the neighboring sites with 1.53-fold at BZ. This change in the total number was not detectable in the other sides of the same retina (BZ3 and PZ3 vs. Z4) (Fig. 5C).

**Fig. 7:**
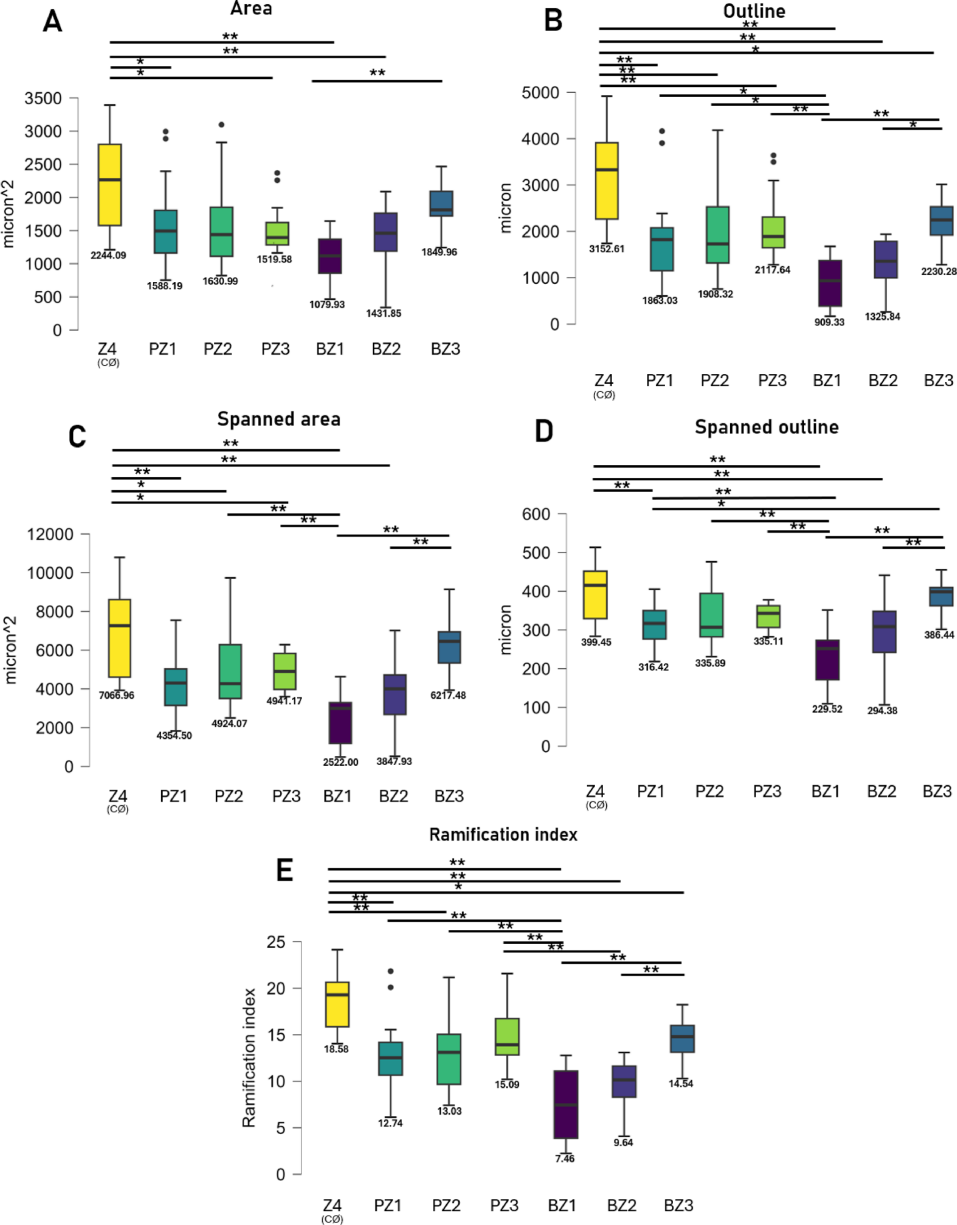
Microglial activation based on automated cell morphologies with MotiQ^32^ based on a sample set of pooled cell images (15–15 cells, random) from all zones (BZ1: SRH, BZ2: neighboring, BZ3: contralateral side from the same retina, PZ1: PBS subretinal injected, PZ2: PBS neighboring, PZ3: PBS injected-contralateral side, Z4: healthy contralateral eye). (**A**) Total occupied area of cells decreasing when activated together with cell outlines (**B**), spanned areas (**C**), and Spanned outlines (**D**). The Ramification index shows the complexity of MG end-feet. ANOVA, Tukey’s post hoc **p < 0.01 *p < 0.05 o = outliers x = mean. Original data available in the Supplementary Dataset.

For objectivity reasons, we tested microglia morphometric changes with MotiQ, an automated microglia morphological toolset^32^. An outlined activation profile was detected even without human supervision, showing a significant decrease in the cellular and spanned areas with a decrease in the ramification indexes of BZ1-3 and PZ1-2 (but not PZ3) when compared to Z4 (Fig. 7). Notably, microglial activation can be detected in both BZ1-3 and PZ1-2 in comparison to Z4. SRH induced a more pronounced activation morphology, indicated by the retraction of the microglial end-feet and the definite decrease in the ramification index averages that follow a regressive tendency (Z4 = 18.58, PZ1 = 12.74, BZ1 = 7.46, Fig. 7E).

### Microglia in the subretinal space

**Fig. 8:**
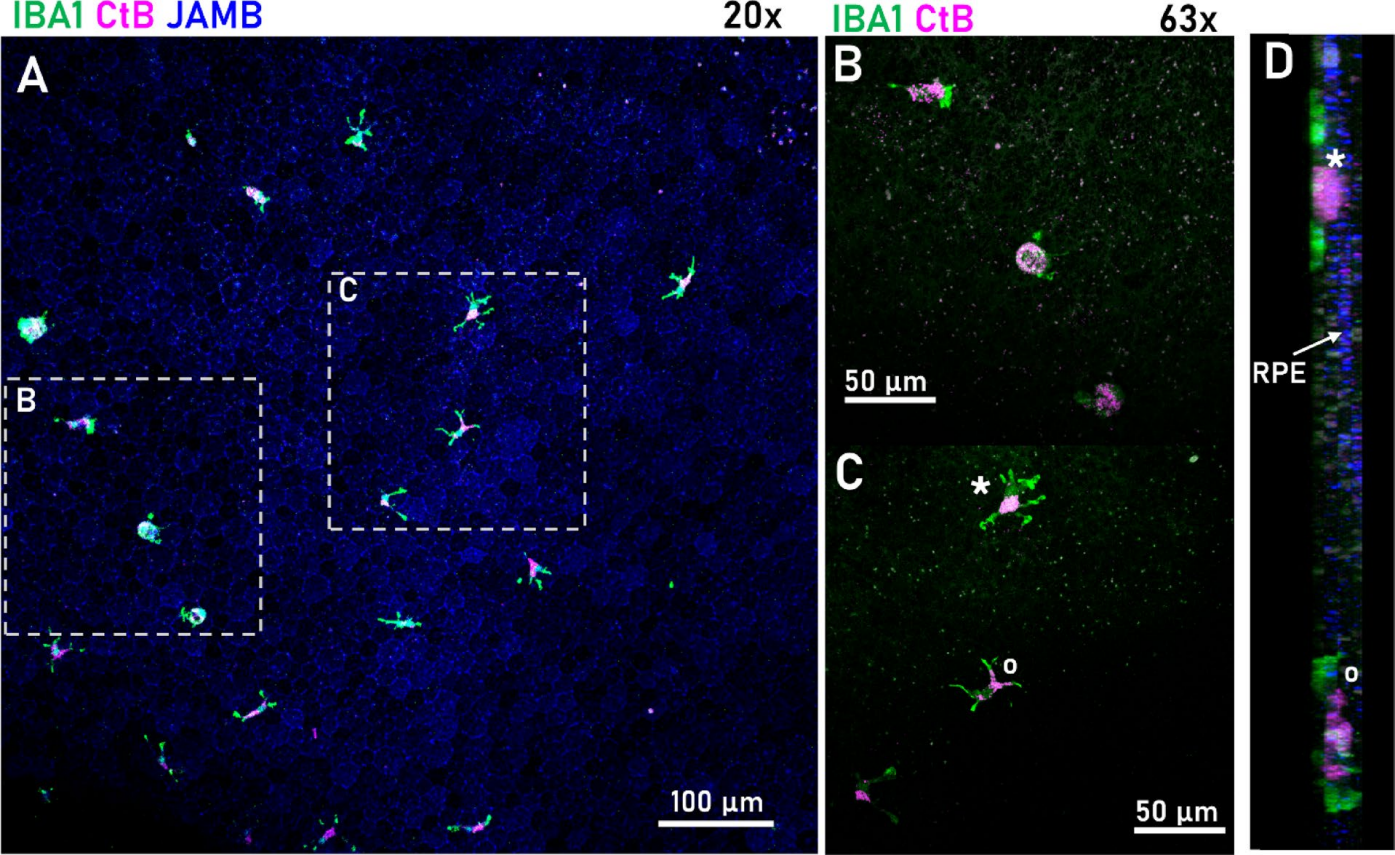
Iba1^+^ cells infiltrated into the subretinal space showed activated morphology in apposition to Z1. (**A**) RPE from SRH-treated animals. **B-C**) High-magnification of the CtB^+^ Iba1^+^ cells. The cells on (**B**) show ellipsoid macrophage morphology, possibly infiltrated macrophages or activated microglia, while most of the Iba1^+^ cells were classified as activated microglia as on panel **C**). **D**) The cells marked in panel C are shown Y-angle rotated in a position to the RPE.

In healthy animals, microglia are normally present only in the inner retina. We examined if SRH attracted inner retinal microglia to the affected subretinal site or induced a macrophage infiltration. Based on the morphological divergence, we regularly observed microglia in direct contact with the RPE layer with only a sparse presence of macrophage-like Iba1^+^ cells (6 macrophage-like morphologies out of 27 Iba1^+^ cells, *n* = 2; Fig. 8). By using the presence of Junctional Adhesion Molecule B (JamB), we marked RPE tight junctions to outline the microglia on the retinal side. Although fewer in number, these cells consistently exhibited activated morphologies, characterized by enlarged and disorganized somata, increased relative soma size, amoeboid and leaf-like structures at the dendritic end-feet. We were unable to find Iba1^+^ cells attached to the RPE in healthy, control retinas, with only the JamB labeling of RPE cells being clearly visible (**Suppl. Figure 1**).

### CtB labeling on and inside microglia

**Fig. 9:**
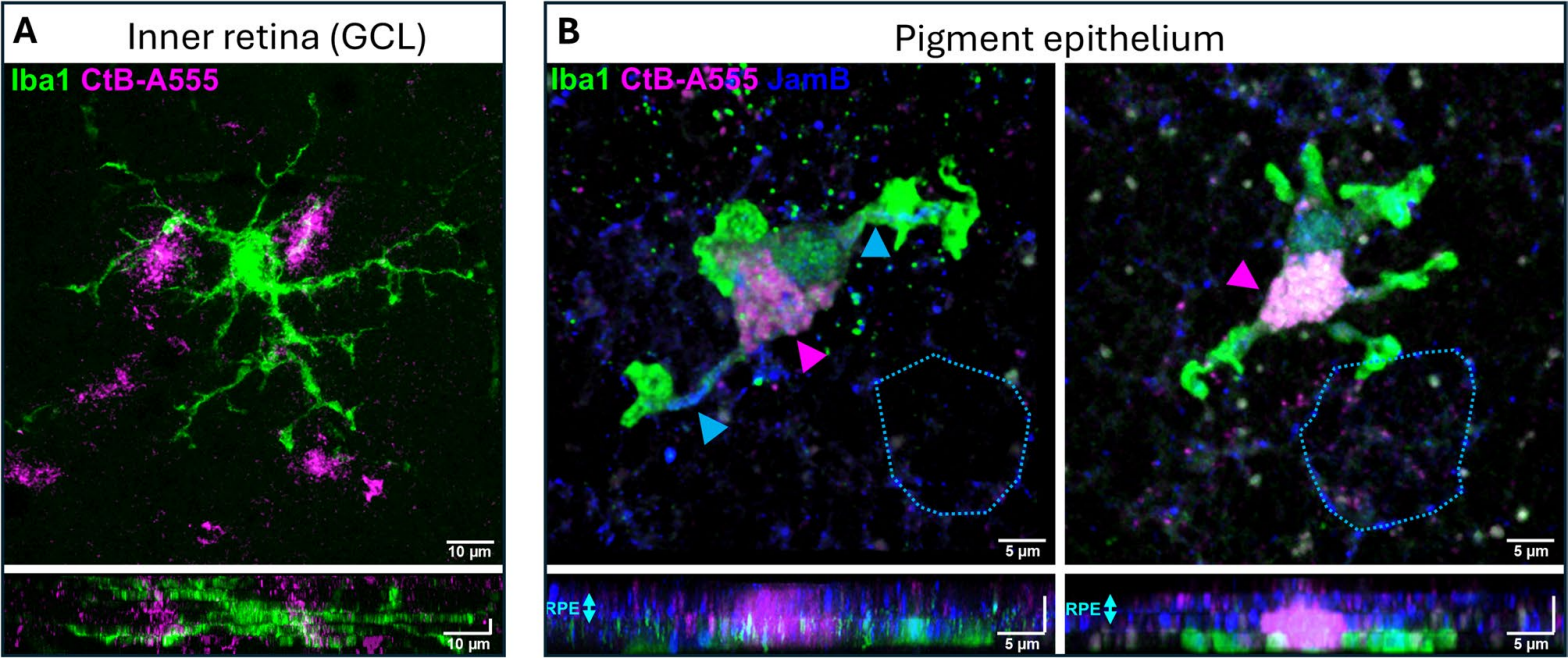
High-resolution images from microglia in the inner retina (A; GCL) and RPE (B). CtB is only internalized by RPE microglia (**B**) but not inner retinal ramified microglia contrary to the direct contact with the CtB-A555 particles of these cells (**A**). In the RPE, Iba1^+^ cells internalized the dye and clearly expressed JamB tight junction protein (blue arrowheads) (**Suppl. Video 1** for rotation of B); JamB is normally expressed only by RPE cells (blue, highlighted with light blue dots).

**Fig. 10:**
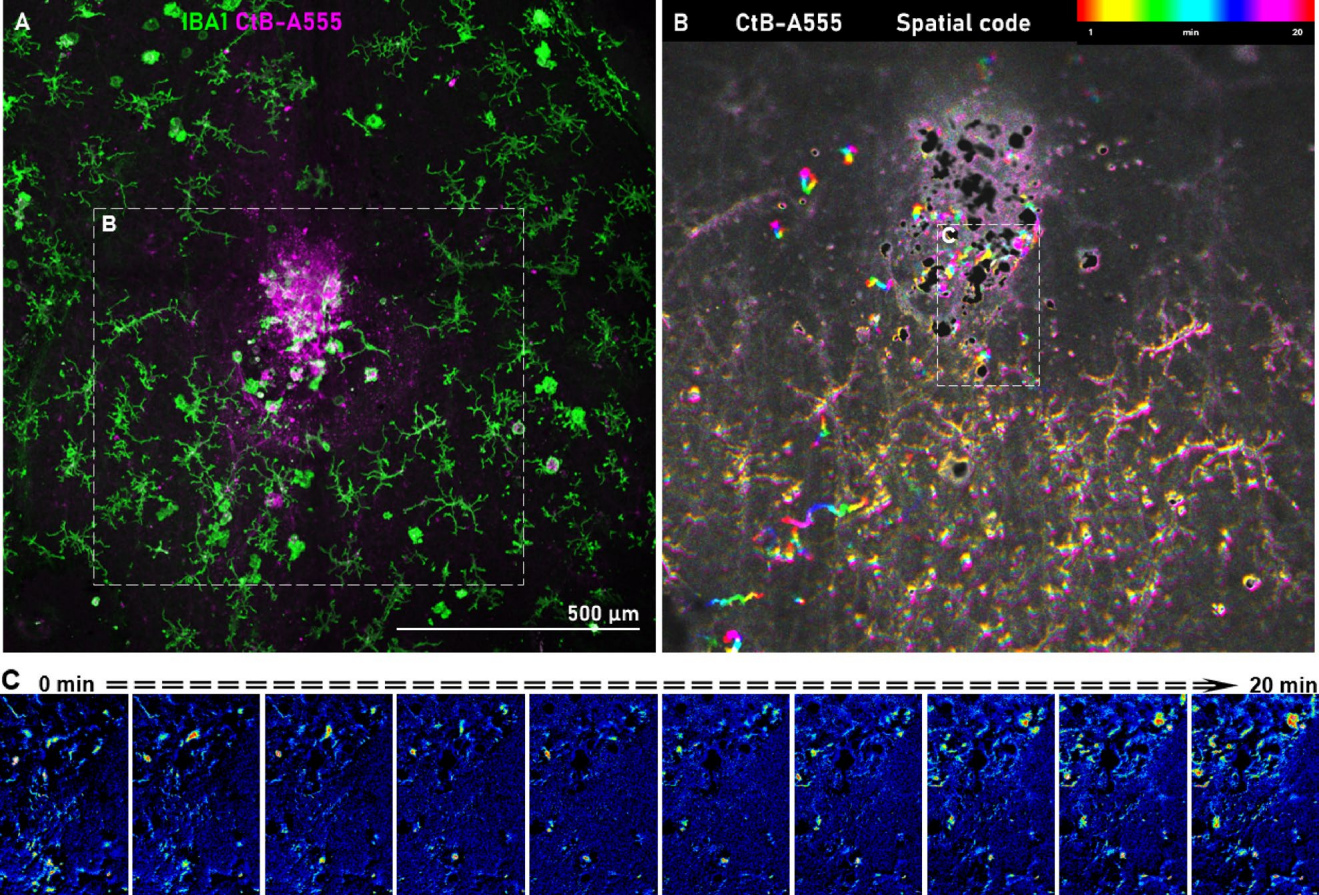
SRH-induced microglial motility in the live retina. (**A**) Ex vivo confocal images from the live imaging sample area (the dashed area outlines the visible area in B). (**B**) Image created by collapsing 81 frames of a 20-minute-long time-lapse recording along the time domain, where colors represent the frames (see the color scale on top). This image sequence was recorded at the SRH injection site, 24 h post-injection with CtB-A555. The presence of rainbow-colored structures reflects the time-dependent change in the location of structures; when the location of a structure is unchanged, the corresponding pixels contain all colors thus they appear white. See also the time-lapse **Suppl. Video 2.** (**C**) Motion detected in the inset during the 20-minutes timelapse video (15 s/frame time-lapse, 4xZ-merged). Each image represents the accumulated movement in 2 min. Color code shows movement activity across frames.

Previous studies suggested that CtB can form direct contact with microglial cell membranes^43^. By using high magnification microscopy, we observed the internalization of CtB-A555 in infiltrated microglia, in the vicinity of the RPE (Fig. 9). Interestingly, this was not the case with the rest of the inner retinal microglia, as we did not detect internalization of CtB particles in these cells. However, their end-feet still exhibited directed movement toward the dye particles (Fig. 9B). This observation was reinforced by the ex vivo live imaging, where we were able to follow the microglia in real-time and observed their change in motility that increased due to inflammatory signals. Microglia appeared highly motile, and they also started breaking down CtB plaques by phagocytosis and transporting them (Fig. 10).

## Discussion

### Novel SRH model

Our method is novel due to the utilization of CtB-A555 co-injection. CtB-conjugates generally serve as retrograde neuronal tracers^44^ that, following endocytosis, are transported back to the Golgi network and the endoplasmic reticulum. In our labeling scheme, CtB-A555 has been used for the first time to help identify the injection site. In contrast to previous models (Table 2), no air or heparin was used to stop the rapid clotting. Instead, the sterile and inert glass injector gives enough working time to perform the SRH injection. The use of smaller, pulled, and therefore heat-sterilized needles protect against reflux (blood or blood-CtB mix) at the injection site^45^. There were no signs of infections, such as redness, purulent discharge, swelling, discomfort, irritation indicated by scratching, photophobia. The use of a thin, hollow microneedle enables a minimally invasive delivery method, as the microneedle only penetrates some hundreds of micrometers into the eyeball and narrows down to 8–12 μm at the tip. This design minimizes mechanical trauma to the retina and reduces the risk of infection^46^.

In microglia, CtB is colocalized either by binding to GM1 ganglioside glycolipid molecules in lipid rafts or was engulfed by microglia and stored intracellularly^43^. In our experiments, we show that the A555 conjugate was similarly localized intracellularly. Previous studies have shown that activated microglial cells can engulf various dyes due to their tissue-specific clearing phagocytic activity^47,48^. Apparently, microglia can internalize CtB molecules as well *via* similar mechanisms and thus its injection combined with fluorescent fundoscopy can be used to visualize cells even in the human eye^49^. Although recently developed adaptive optics fundoscopy offers a dye-free, minimally invasive method for visualizing microglia in both mouse and human eyes—enabling direct, live retinal imaging^50–55^ our CtB-conjugate coinjection method remains a cost-efficient alternative for laboratories with limited or no access to such advanced equipment. Finally, even though it served different aims in our study, CtB could have exerted anti-inflammatory effects as well^56,57^ thereby further decreasing the risk of inflammation in the experiments.

### Microglia activation in SRH

Microglia are resident macrophages of the retina that perform surveillance and neuroprotection against pathophysiological insults^58^. In the healthy retina, they are quiescent and confined to the GCL and the plexiform layers. When microglia get activated and migrate to the location of the insult, they may appear among photoreceptor outer segments or RPE in retinitis pigmentosa and age-related macular degeneration, respectively^59^. Similarly, we observed that after SRH, microglia appear in the vicinity of the RPE, with the majority of these ectopically localized cells exhibiting morphological features characteristic of an activated microglial phenotype.

Previous studies limited their examinations to the outer retina (RPE and photoreceptors) whereas possible parallel inner retinal changes have been largely overlooked. In this present study we extended our analysis to include inner retinal areas as well and identified a widespread SRH treatment induced microglia activation. This suggests that SRH initiated a complex chemotactic activation of blood-related factors, including fibrin and albumin. When these factors appear in the extravasal space^60^ and diffuse to inner tissue compartments, they activate microglia, which subsequently migrate towards the blood clot to facilitate clearance. By performing this radial migration, microglia must traverse photoreceptor inner and outer segments before ultimately reaching the RPE.

To our knowledge, we performed PBS subretinal injections for the first time. We checked the integrity of inner retinal vasculature with albumin labeling. Compared to PBS injection, the number of activated microglia was only slightly elevated in the SRH, however, a more pronounced reduction in the number of non-activated microglia was observed, indicating an overall decrease in total microglial counts near the SRH site of injection (Fig. 5C). We hypothesize that an increased number of cells are interacting with blood-associated components, leading to their internalization of these materials and subsequent differentiation into macrophage-like cells that exit the retina via the bloodstream^25^. This proposed mechanism is supported by observed morphological changes in SRH cells, which exhibit more amoeboid, macrophage-like characteristics, such as reduced cell area and decreased ramification indices (Fig. 7). This phenomenon requires further research, probably by using our CtB-A555 labeling, which allows the exiting phagocytic cells to be detected in the blood retrieved from the mice.

In our study, we observed both microglia that internalized CtB-A555 (Fig. 8) as well as also microglia with no sign of internalized particles. This indicates that a population of microglia participated in the clearance of the dye through phagocytosis while the rest of them were only activated without transforming into phagocytic cells. However, it is uncertain whether these two functionally different populations exist or phagocytotic transformation was just delayed for some of the cells. In a previous study, the accumulation of fluorescent carbocyanine vital dyes in microglia demonstrated the phagocytosis of dying neurons^61^. Here, we showed that CtB-A555 molecules can be endocytosed by microglia from the extracellular milieu (Fig. 8).

CtB has potential anti-inflammatory effects as previously shown by Zhang et al.^56^. 24 h after ischemia, CtB downregulated the levels of proinflammatory cytokines and microglia/macrophage transformation. Downregulation of MGs through enhancement of CD200-CD200R interaction or CX3CR1 (fractaline-1) could be a potential avenue for therapeutic intervention^16,19^.

Based on microglia morphological characteristics, we did not observe any indication of this latter CtB-induced effect as microglia counts of simple blood injections and CtB/blood coinjected samples appeared similar (data not shown). Microglia activated by SRH can release a number of proinflammatory cytokines that can induce inflammation and corresponding subsequent tissue degeneration^34^. Therefore, any inflammatory changes observed in our experiments are rather induced by the SRH and corresponding microglia activation and are largely independent of the presence of coinjected CtB.

### Bordering zones and localization

Our results indicate that SRH is predominantly confined to a defined Z1 region encompassing the injection site and the primary insult caused by the blood clot. Surprisingly, microglia in the Z2 area were activated as well contrary to the fact that they were not in direct contact with the blood clot itself. Z2 microglia may get informed about the insult through proinflammatory factors^34^ either originating from the clot and/or released by activated microglia of the Z1 site. Contrary, we have not detected activation or changes in the number of microglia in the non-affected Z3 and Z4 sites (Figs. 5 and 6). Therefore, we hypothesize that SRH is limited to the Z1 and Z2 sites while the rest of the tissue remains intact.

## Conclusions

SRH can be a devastating disease affecting vision. Our new model can help identify new treatment methods while enabling the monitoring of microglia with dye-conjugated CtB. The results show that overlaying inner retinal microglia are activated even if blood is injected between the photoreceptors and the RPE in contrast to non-treated controls and also to less extent but to PBS controls as well. Activated microglia bind CtB-A555 but are less likely to phagocytose it while microglial and infiltrated macrophages at the RPE will internalize the dye. This internalized CtB dye, and the morphological changes of microglia can be visualized in the living tissue for the monitoring of the microglial activation process. Moreover, in this model, the borders of the SRH site can be precisely demarcated thus allowing for the development of future solutions to avoid retinal cell death or to use microglia as biomarkers during pathological changes.

## Electronic supplementary material

Below is the link to the electronic supplementary material.

Supplementary Material 1

Supplementary Material 2

Supplementary Material 3

Supplementary Material 4

Supplementary Material 5

## Data Availability

The datasets are available as “Supplementary Dataset”. Original images used and/or analyzed during the current study are available from the corresponding author upon reasonable request.

## Abbreviations

A555: Alexa-555 (fluorescent dye)
Am: Amacrine cell
AMD: age-related macular degeneration
AO: adaptive optics
AS: Astrocyte
BC: Bipolar cell
C: Cone
cLSM: confocal laser scanning microscope
CNS: central nervous system
CTA: Blocking solution containing 5% Chemiblocker, TritonX-100, 0.05% Na-azide in PBS
CtB: Cholera toxin subunit B
DL: Deep layer (of microglia)
GC: Ganglion cell
GCL: Ganglion cell layer
HC: Horizontal cell
IBA1: Ionized calcium-binding adapter molecule 1
INL: Inner Nuclear Layer
IPL: Inner Plexiform Layer
JAM-B: Junctional adhesion molecule B
NFL: Neurofilament Layer
OCT: optical coherence tomography
ONL: Outer Nuclear Layer
OPL: Outer Plexiform Layer
OSL: Outer Segment Layer
PBS: Phosphate buffered saline solution
RPE: retinal pigment epithelium
RT: room temperature
SL: Superficial Layer (of microglia)
SRH: Subretinal hemorrhage
Z1-4: zone 1-4
