# Correction: Local microglial activation induced and labeled in the retina in a novel subretinal hemorrhage mouse model

**DOI:** 10.1038/s41598-025-18785-2

**Published:** 2025-09-12

**Authors:** Boglárka Balogh, Marietta Zille, Gergely Szarka, Loretta Péntek, Anett Futácsi, Béla Völgyi, Tamás Kovács-Öller

**Affiliations:** 1 Szentágothai Research Centre, University of Pécs, Pécs, Hungary; 2 Department of Neurobiology, Institute of Biology, Faculty of Sciences, University of Pécs, Pécs, Hungary; 3 Division of Pharmacology and Toxicology, Department of Pharmaceutical Sciences, University of Vienna, Vienna, Austria; 4 NEURON-066 Rethealthsi Research Group, Pécs, Hungary; 5 Imaging Core Facility, Szentágothai Research Centre, University of Pécs, Pécs, Hungary; 6 Medical School, University of Pécs, Pécs, Hungary

Correction to: *Scientific Reports* 10.1038/s41598-025-09007-w, published online 10 July 2025

The original version of this Article contained errors in Figures 4 and 9 due to a technical issue during production. Panels A,B,C and D were duplicated in Figure 4, and panels A and B were duplicated in Figure 9. The original Figure 4 and 9 and accompanying legend appear below.

The original Article has been corrected.

**Fig. 4:**
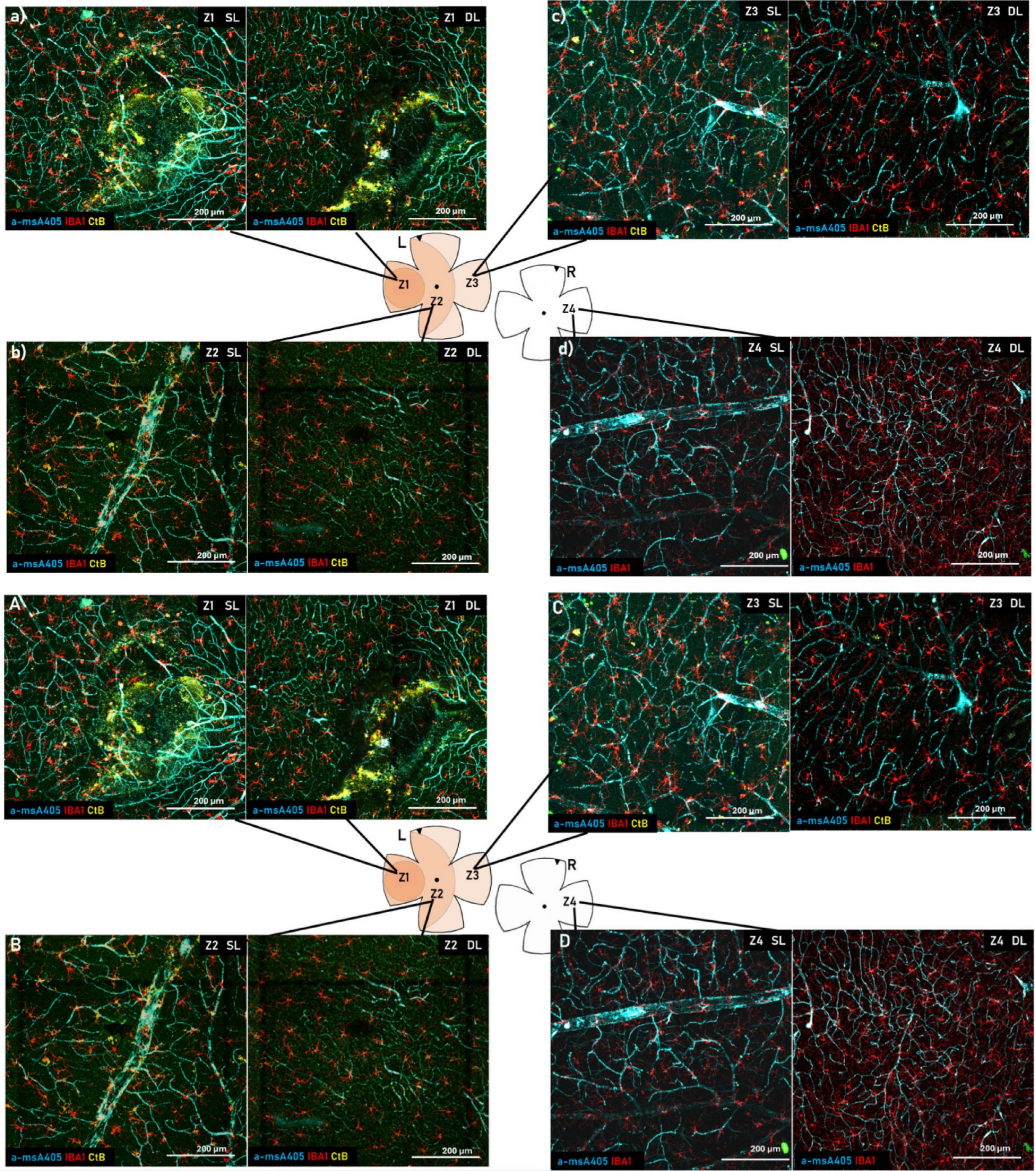
Example microscopic images of the SRH site and the surrounding areas. (**A**) SRH site (Z1) at the level of the superficial layer (left) and the deep layer (right). (**B**) Neighboring zone (Z2) superficial layer (left) and the deep layer (right). (**C**) Z3, the farthest zone from the SRH site at the level of the superficial layer (left) and the deep layer (right). (**D**) Control retina (Z4; contralateral eye) at the level of the superficial layer (left) and the deep layer (right). All images are 20 × magnification confocal images.

**Fig. 9:**
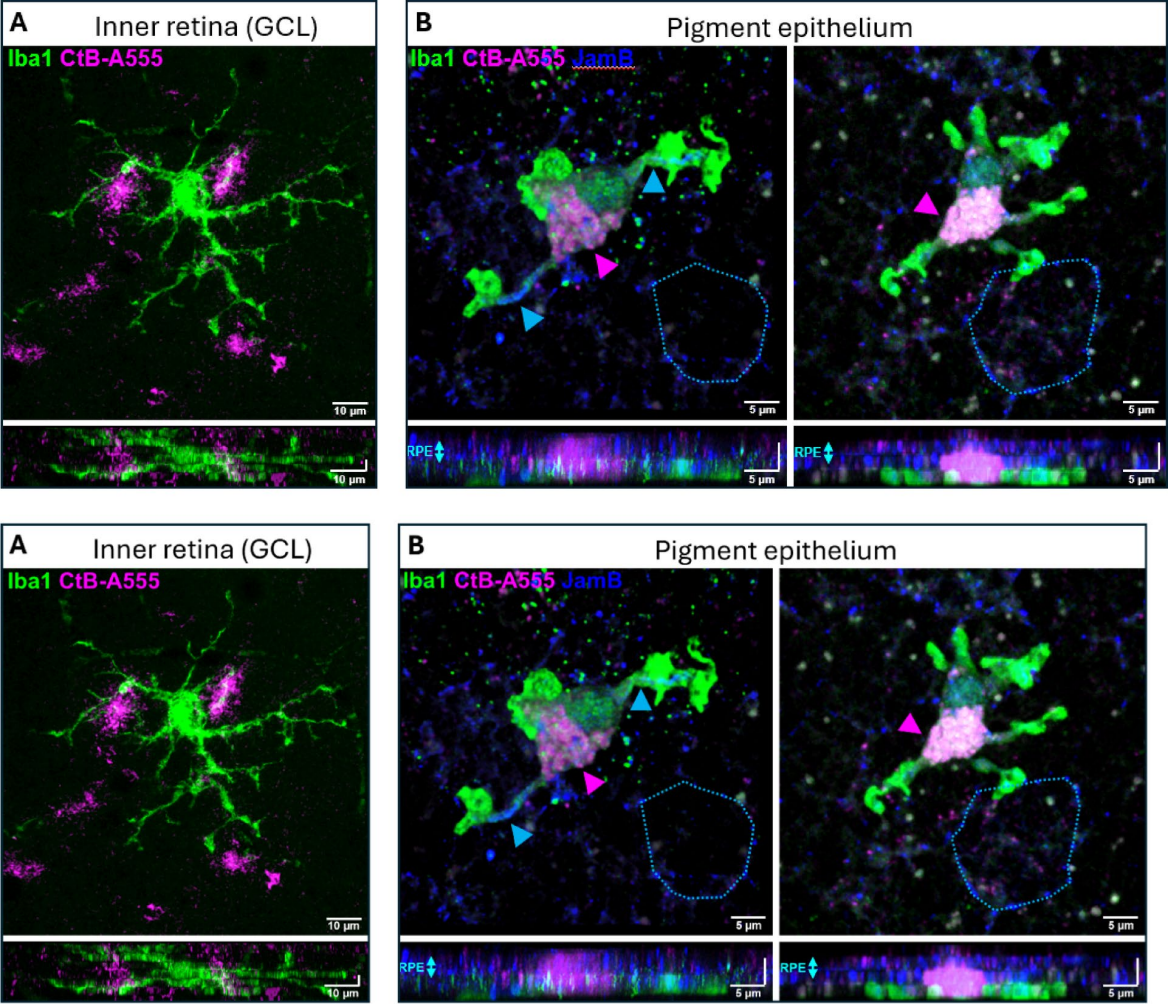
High-resolution images from microglia in the inner retina (A; GCL) and RPE (B). CtB is only internalized by RPE microglia (**B**) but not inner retinal ramified microglia contrary to the direct contact with the CtB-A555 particles of these cells (**A**). In the RPE, Iba1^+^ cells internalized the dye and clearly expressed JamB tight junction protein (blue arrowheads) (**Suppl. Video 1** for rotation of B); JamB is normally expressed only by RPE cells (blue, highlighted with light blue dots).

