# Supplementary figures and images for: Local microglial activation induced and labeled in the retina in a novel subretinal hemorrhage mouse model
Source: Sci Rep. 2025 Jul 10;15:24804. doi: 10.1038/s41598-025-09007-w (PMC12246455; doi:10.1038/s41598-025-09007-w)

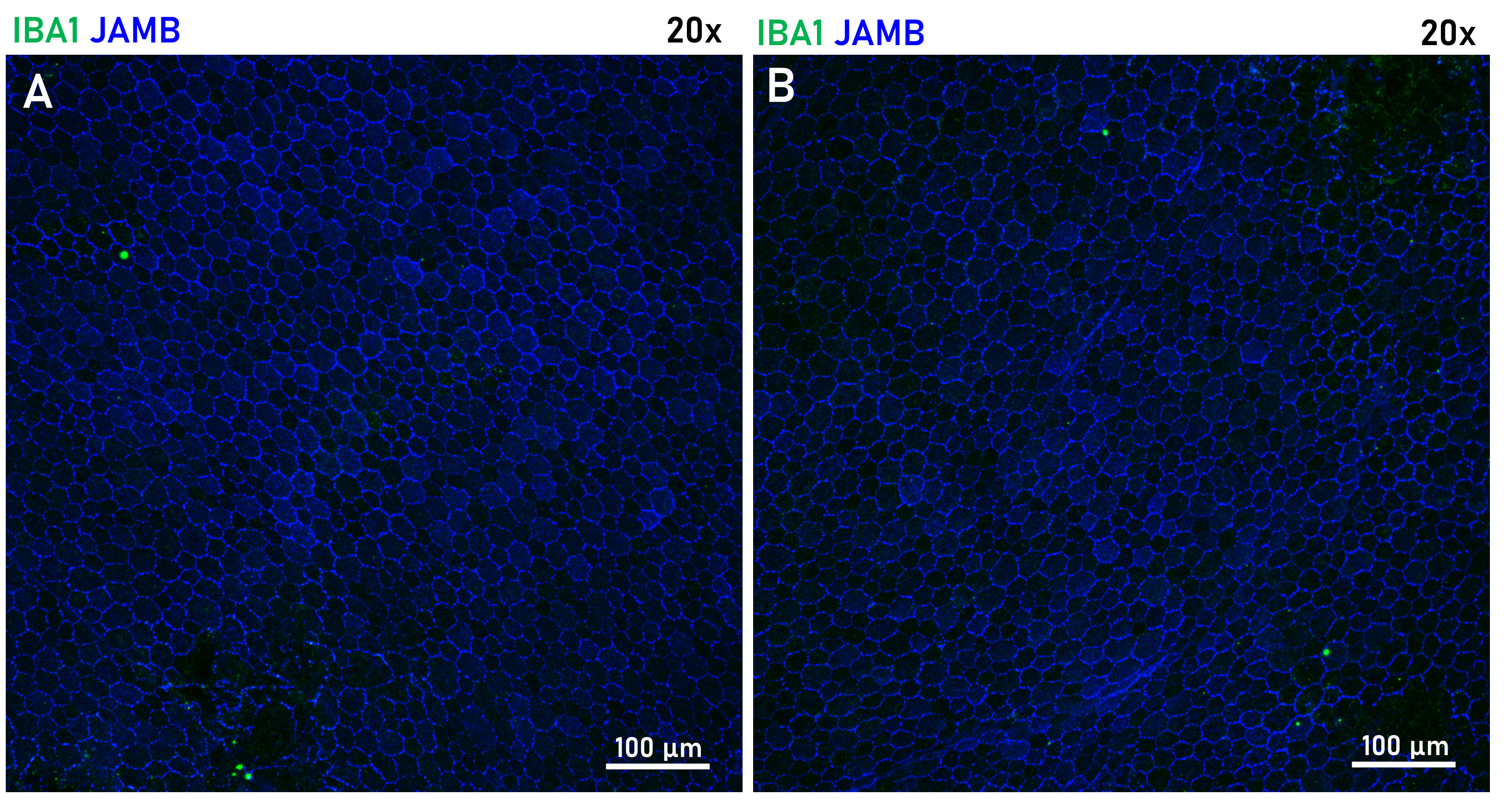

Supplement: Supplementary file 1 — Supplementary Material 1 [file 41598_2025_9007_MOESM1_ESM.tif]
