# Supplementary material for: Local microglial activation induced and labeled in the retina in a novel subretinal hemorrhage mouse model
Source: Sci Rep. 2025 Jul 10;15:24804. doi: 10.1038/s41598-025-09007-w (PMC12246455; doi:10.1038/s41598-025-09007-w)
Supplement: Supplementary file 4 — Supplementary Material 4 [file 41598_2025_9007_MOESM4_ESM.pdf]

Local Microglial Activation Induced and Labeled in the Retina in a Novel Subretinal Hemorrhage Mouse Model

Boglárka Balogh[1,2], Marietta Zille[3], Gergely Szarka[1,2,4,5,6], Loretta Péntek[1,2], Anett Futácsi[1,4,5,6] Béla Völgyi[1,2,4,5,6], Tamás Kovács-Öller[1,2,4,5,6,*]

[1] Szentágothai Research Centre, University of Pécs, Pécs, Hungary

[2] Department of Neurobiology, Institute of Biology, Faculty of Sciences, University of Pécs, Pécs, Hungary

[3] Division of Pharmacology and Toxicology, Department of Pharmaceutical Sciences, University of Vienna, Vienna, Austria

[4] NEURON-066 Rethealthsi Research Group, Pécs, Hungary

[5] Imaging Core Facility, Szentágothai Research Centre, University of Pécs, Pécs, Hungary

[6] Medical School, University of Pécs, Pécs, Hungary

* Correspondence: kovacs-oller.tamas@pte.hu

Supplemental figures

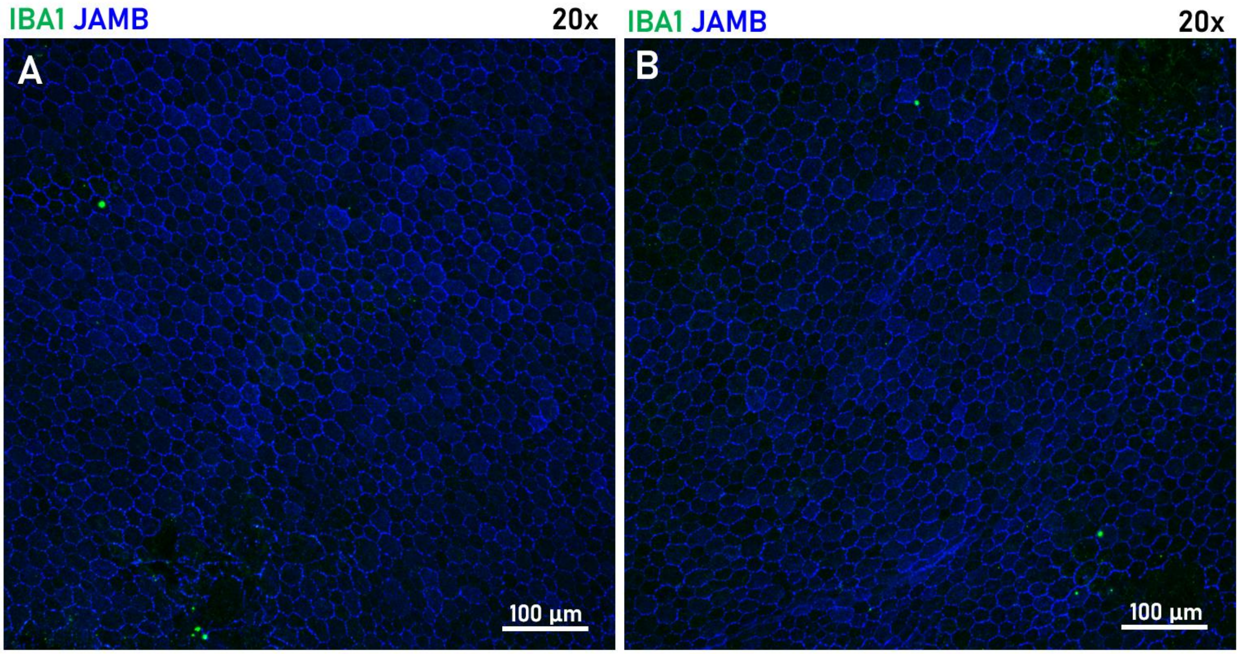

Supplemental Figure 1. *Control RPE without the presence of Iba1^{+} cells.*

Supplemental Video Legends

Supplementary Video 1: *High-resolution 3D rotation of microglia from the RPE (B)*. *X-axis rotation from CtB-A555 (red) labeled Iba1^{+} (green) cell in direct contact with JamB+ tight junctions of the RPE (blue). Original Z-merge: Figure 8B, right.*

Supplementary Video 1: Time-lapse imaging of *SRH-induced microglial motility, labeled by CtB-A555 in the live retina.* *Live retinal video created from 81 frames (with 2*Z-binning) of a 20-minute-long time-lapse recording from perfused ex vivo retinal confocal images representing the same area as in Figure 9 A and B. CtB-A555 dye labeled cells are clearlüy visible 24-hours post-SRH injection and individual dye particles are actively moved away from the SRH site by microglial cells. Fire LUT (Lookup table) mode was applied for visualization. Image was captured using the 543 nm laser line and the corresponding A555-specific filter set.*
