# Supplementary material for: Local microglial activation induced and labeled in the retina in a novel subretinal hemorrhage mouse model
Source: Sci Rep. 2025 Jul 10;15:24804. doi: 10.1038/s41598-025-09007-w (PMC12246455; doi:10.1038/s41598-025-09007-w)
Supplement: Supplementary file 5 — Supplementary Material 5 [file 41598_2025_9007_MOESM5_ESM.pdf]

Local Microglial Activation Induced and Labeled in the Retina in a Novel Subretinal Hemorrhage Mouse Model

Boglárka Balogh[1,2], Marietta Zille[3], Gergely Szarka[1,2,4,5,6], Loretta Péntek[1,2], Anett Futácsi[1,4,5,6] Béla Völgyi[1,2,4,5,6], Tamás Kovács-Öller[1,2,4,5,6,*]

[1] Szentágothai Research Centre, University of Pécs, Pécs, Hungary

[2] Department of Neurobiology, Institute of Biology, Faculty of Sciences, University of Pécs, Pécs, Hungary

[3] Division of Pharmacology and Toxicology, Department of Pharmaceutical Sciences, University of Vienna, Vienna, Austria

[4] NEURON-066 Rethealthsi Research Group, Pécs, Hungary

[5] Imaging Core Facility, Szentágothai Research Centre, University of Pécs, Pécs, Hungary

[6] Medical School, University of Pécs, Pécs, Hungary

* Correspondence: kovacs-oller.tamas@pte.hu

Supplemental tables

Supplementary Table 1. Activated microglia numbers in the different zones, and categorized by injection type. Dataset used for Figure 5 and Figure 6.

	Activated microglia numbers							
	Superficial layer (SL)				Deep layer (DL)			
	**Z1**	**Z2**	**Z3**	**Z4**	**Z1**	**Z2**	**Z3**	**Z4**
	40	11	17	13	42	39	22	4
	25	28	9	13	15	26	7	6
**CtB**	7	6	3	7	1	16	4	3
	8	11	3	6	4	18	10	8
	5	20	2	[redacted]	8	22	9	[redacted]
	50	19	8	12	53	16	5	4
	36	29	3	11	15	31	7	1
**Blood**	29	15	17	8	19	11	3	6
	29	22	2	3	16	25	3	6
	[redacted]	[redacted]	[redacted]	13	[redacted]	[redacted]	[redacted]	10
	18	23	19	5	17	24	14	7
**PBS**	21	11	14	9	24	20	17	4
	15	19	8	13	25	15	12	4
	29	17	13	12	26	13	7	8
	34	14	20	10	15	6	7	1

Supplementary Table 2. Non-activated microglia numbers in the different zones, categorized by injection type. Dataset used for Figure 5 and Figure 6.

	Non-activated microglia numbers							
	Superficial layer (SL)				Deep layer (DL)			
	Z1	Z2	Z3	Z4	Z1	Z2	Z3	Z4
CtB	50	15	45	49	57	13	40	61
	15	20	40	47	19	18	45	63
	0	10	29	32	0	12	44	39
	4	11	49	43	16	24	61	63
	8	8	40		9	15	53	
Blood	43	25	35	46	34	20	33	48
	28	29	48	48	35	18	40	53
	14	5	24	38	4	5	7	41
	33	30	34	35	41	28	52	50
PBS				47				46
	26	29	35	45	32	40	63	53
	27	38	51	40	47	53	49	64
	14	58	41	47	24	54	50	71
	39	47	66	57	40	57	74	71
	32	34	75	40	42	31	59	54

Supplementary Table 3. Total numbers in different zones, categorized by injection type. Dataset used for Figure 5 and Figure 6.

	Total microglia numbers			
	Z1	Z2	Z3	Z4
CtB	189	78	124	127
	74	92	101	129
	8	44	80	81
	32	64	123	120
	30	65	104	
Blood	180	80	81	110
	114	107	98	113
	66	36	51	93
	119	105	91	94
PBS	93	116	131	116
	119	122	131	110
	78	146	111	117
	134	134	160	135
	123	85	161	148
				105

Supplementary Table 4. Microglia numbers in the retinal pigment epithelium. Dataset referenced in main text Line283-284.

		Total microglia	Macrophage-like	Microglia-like
CtB	RPE1	2	2	0
	RPE2	24	3	21
Control	RPE1	0	0	0
	RPE2	0	0	0

Supplementary Table 5. Kruskal-Wallis test results for activated microglia numbers in the different zones with different SRH injection types. Shows there is no difference in the activated microglia numbers after using the tracer molecule with the injections. Statistical result not shown in main text.

Hypothesis Test Summary

	Null Hypothesis	Test	Sig.	Decision
1	The medians of Zone1 are the same across categories of InjectionType.	Independent-Samples Median Test	,206[1,2]	Retain the null hypothesis.
3	The medians of Zone2 are the same across categories of InjectionType.	Independent-Samples Median Test	1,000[1,2]	Retain the null hypothesis.
5	The medians of Zone3 are the same across categories of InjectionType.	Independent-Samples Median Test	1,000[1,2]	Retain the null hypothesis.

Asymptotic significances are displayed. The significance level is ,05.

[1]Exact significance is displayed for this test.

[2]Fisher Exact Sig.

Supplementary Table 6. Kruskal-Wallis test results for non-activated microglia numbers in the different zones with different SRH injection types. Shows there is no difference in the activated microglia numbers after using the tracer molecule with the injections. Statistical result not shown in main text.

Hypothesis Test Summary

	Null Hypothesis	Test	Sig.	Decision
1	The medians of Zone1 are the same across categories of Injection.	Independent-Samples Median Test	,206[1,2]	Retain the null hypothesis.
3	The medians of Zone2 are the same across categories of Injection.	Independent-Samples Median Test	,206[1,2]	Retain the null hypothesis.
5	The medians of Zone3 are the same across categories of Injection.	Independent-Samples Median Test	1,000[1,2]	Retain the null hypothesis.

Asymptotic significances are displayed. The significance level is ,05.

[1]Exact significance is displayed for this test.

[2]Fisher Exact Sig.

Supplementary Table 7.Kruskal-Wallis test statistics for activated microglia numbers in the different zones. Result used in Figure 5B.

Factor	Statistic	df	p
Zone	29.601	6	< .001

Supplementary Table 8. Dunn's post hoc comparisions for activated microglia numbers in the different zones.Results used in Figure 5B.

Comparison	z	W_i	W_j	r_{rb}	p
BZ1 - BZ2	−0.745	35.833	41.556	0.012	0.456
BZ1 - BZ3	2.835	35.833	14.056	0.605	0.005**
BZ1 - PZ1	−1.041	35.833	45.300	0.067	0.298
BZ1 - PZ2	0.070	35.833	35.200	0.244	0.944
BZ1 - PZ3	0.609	35.833	30.300	0.333	0.543
BZ1 - Z4	2.900	35.833	15.643	0.484	0.004**
BZ2 - BZ3	3.580	41.556	14.056	0.901	< .001***
BZ2 - PZ1	−0.412	41.556	45.300	0.178	0.680
BZ2 - PZ2	0.699	41.556	35.200	0.356	0.484
BZ2 - PZ3	1.238	41.556	30.300	0.644	0.216
BZ2 - Z4	3.722	41.556	15.643	0.984	< .001***
BZ3 - PZ1	−3.437	14.056	45.300	0.956	< .001***
BZ3 - PZ2	−2.326	14.056	35.200	0.800	0.020*
BZ3 - PZ3	−1.787	14.056	30.300	0.733	0.074
BZ3 - Z4	−0.228	14.056	15.643	0.206	0.820
PZ1 - PZ2	0.980	45.300	35.200	0.760	0.327
PZ1 - PZ3	1.455	45.300	30.300	1.000	0.146
PZ1 - Z4	3.493	45.300	15.643	1.000	< .001***
PZ2 - PZ3	0.475	35.200	30.300	0.400	0.635
PZ2 - Z4	2.303	35.200	15.643	0.957	0.021*
PZ3 - Z4	1.726	30.300	15.643	0.914	0.084

* p < .05, ** p < .01, *** p < .001

Note. Rank-biserial correlation based on individual Mann-Whitney tests.

Supplementary Table 9. Kruskal-Wallis test statistics for non-activated microglia numbers in the different zones. Results not shown in main text.

Factor	Statistic	df	p
Zone	33.560	6	< .001

Supplementary Table 10. Dunn's post hoc comparisions for non-activated microglia numbers in the different zones. Results not shown in main text.

Comparison	z	W_i	W_j	r_{rb}	p
BZ1 - BZ2	0.708	15.667	10.222	0.062	0.479
BZ1 - BZ3	−1.843	15.667	29.833	0.605	0.065
BZ1 - PZ1	−0.641	15.667	21.500	0.378	0.521
BZ1 - PZ2	−2.082	15.667	34.600	0.644	0.037*
BZ1 - PZ3	−3.358	15.667	46.200	0.867	< .001***
BZ1 - Z4	−3.729	15.667	41.643	0.810	< .001***
BZ2 - BZ3	−2.552	10.222	29.833	0.877	0.011*
BZ2 - PZ1	−1.240	10.222	21.500	0.822	0.215
BZ2 - PZ2	−2.681	10.222	34.600	1.000	0.007**
BZ2 - PZ3	−3.956	10.222	46.200	1.000	< .001***
BZ2 - Z4	−4.511	10.222	41.643	1.000	< .001***
BZ3 - PZ1	0.916	29.833	21.500	0.511	0.359
BZ3 - PZ2	−0.524	29.833	34.600	0.200	0.600
BZ3 - PZ3	−1.800	29.833	46.200	0.822	0.072
BZ3 - Z4	−1.695	29.833	41.643	0.579	0.090
PZ1 - PZ2	−1.270	21.500	34.600	0.520	0.204
PZ1 - PZ3	−2.395	21.500	46.200	1.000	0.017*
PZ1 - Z4	−2.371	21.500	41.643	0.900	0.018*
PZ2 - PZ3	−1.125	34.600	46.200	0.480	0.261
PZ2 - Z4	−0.829	34.600	41.643	0.329	0.407
PZ3 - Z4	0.537	46.200	41.643	0.271	0.592

* p < .05, ** p < .01, *** p < .001

Note. Rank-biserial correlation based on individual Mann-Whitney tests.

Supplementary Table 11. Kruskal-Wallis test statistics for total microglia numbers in the different zones. Result used in Figure 5C.

Factor	Statistic	df	p
Zone	18.941	6	0.004

Supplementary Table 12. Kruskal-Wallis test statistics for activated microglia numbers in the SL of different zones. Results used in Figure 6A.

Factor	Statistic	df	p
Zone	22.511	6	< .001

Supplementary Table 13. Dunn's post hoc comparisions for activated microglia numbers in the SL of different zones. Results used in Figure 6A.

Comparison	z	W_i	W_j	r_{rb}	p
BZ1 - BZ2	0.499	38.500	34.667	0.309	0.618
BZ1 - BZ3	3.270	38.500	13.389	0.716	0.001**
BZ1 - PZ1	−0.583	38.500	43.800	0.111	0.560
BZ1 - PZ2	0.385	38.500	35.000	0.333	0.700
BZ1 - PZ3	0.793	38.500	31.300	0.356	0.428
BZ1 - Z4	2.797	38.500	19.036	0.484	0.005**
BZ2 - BZ3	2.771	34.667	13.389	0.753	0.006**
BZ2 - PZ1	−1.005	34.667	43.800	0.378	0.315
BZ2 - PZ2	−0.037	34.667	35.000	0.089	0.971
BZ2 - PZ3	0.371	34.667	31.300	0.244	0.711
BZ2 - Z4	2.246	34.667	19.036	0.611	0.025*
BZ3 - PZ1	−3.347	13.389	43.800	0.911	< .001***
BZ3 - PZ2	−2.379	13.389	35.000	0.778	0.017*
BZ3 - PZ3	−1.971	13.389	31.300	0.667	0.049*
BZ3 - Z4	−0.811	13.389	19.036	0.373	0.417
PZ1 - PZ2	0.854	43.800	35.000	0.520	0.393
PZ1 - PZ3	1.213	43.800	31.300	0.680	0.225
PZ1 - Z4	2.918	43.800	19.036	1.000	0.004**
PZ2 - PZ3	0.359	35.000	31.300	0.200	0.719
PZ2 - Z4	1.881	3			[illegible]
PZ3 - Z4	1.445	31.300	19.036	0.671	0.148

* p < .05, ** p < .01, *** p < .001

Note. Rank-biserial correlation based on individual Mann-Whitney tests.

Supplementary Table 14. Kruskal-Wallis test statistics for non-activated microglia numbers in the SL of different zones. Results used in Figure 6B.

Factor	Statistic	df	p
Zone	29.808	6	< .001

Supplementary Table 15. Dunn's post hoc comparisions for non-activated microglia numbers in the SL of different zones. Results used in Figure 6B.

Comparison	z	W_i	W_j	r_{rb}	p
BZ1 - BZ2	0.868	17.444	10.778	0.086	0.386
BZ1 - BZ3	−1.952	17.444	32.444	0.556	0.051
BZ1 - PZ1	−0.149	17.444	18.800	0.178	0.881
BZ1 - PZ2	−1.898	17.444	34.700	0.644	0.058
BZ1 - PZ3	−3.097	17.444	45.600	0.822	0.002**
BZ1 - Z4	−3.183	17.444	39.607	0.690	0.001**
BZ2 - BZ3	−2.820	10.778	32.444	0.889	0.005**
BZ2 - PZ1	−0.882	10.778	18.800	0.600	0.378
BZ2 - PZ2	−2.631	10.778	34.700	0.933	0.009**
BZ2 - PZ3	−3.830	10.778	45.600	1.000	< .001***
BZ2 - Z4	−4.140	10.778	39.607	1.000	< .001***
BZ3 - PZ1	1.501	32.444	18.800	0.644	0.133
BZ3 - PZ2	−0.248	32.444	34.700	0.067	0.804
BZ3 - PZ3	−1.447	32.444	45.600	0.622	0.148
BZ3 - Z4	−1.029	32.444	39.607	0.349	0.304
PZ1 - PZ2	−1.542	18.800	34.700	0.680	0.123
PZ1 - PZ3	−2.600	18.800	45.600	0.920	0.009**
PZ1 - Z4	−2.450	18.800	39.607	0.900	0.014*
PZ2 - PZ3	−1.057	34.700	45.600	0.520	0.290
PZ2 - Z4	−0.578	34.700	39.607	0.229	0.563
PZ3 - Z4	0.706	45.600	39.607	0.357	0.480

* p < .05, ** p < .01, *** p < .001

Note. Rank-biserial correlation based on individual Mann-Whitney tests.

Supplementary Table 16. Kruskal-Wallis test statistics for activated microglia numbers in the DL of different zones. Results used in Figure 6C.

Factor	Statistic	df	p
Zone	29.899	6	< .001

Supplementary Table 17. Dunn's post hoc comparisions for activated microglia numbers in the DL of different zones. Results used in Figure 6C.

Comparison	z	W_i	W_j	r_{rb}	p
BZ1 - BZ2	−1.505	32.944	44.500	0.358	0.132
BZ1 - BZ3	1.809	32.944	19.056	0.444	0.070
BZ1 - PZ1	−1.294	32.944	44.700	0.378	0.196
BZ1 - PZ2	−0.171	32.944	34.500	0.022	0.864
BZ1 - PZ3	0.489	32.944	28.500	0.289	0.625
BZ1 - Z4	2.795	32.944	13.500	0.619	0.005**
BZ2 - BZ3	3.315	44.500	19.056	0.889	< .001***
BZ2 - PZ1	−0.022	44.500	44.700	0.067	0.982
BZ2 - PZ2	1.101	44.500	34.500	0.511	0.271
BZ2 - PZ3	1.762	44.500	28.500	0.778	0.078
BZ2 - Z4	4.456	44.500	13.500	1.000	< .001***
BZ3 - PZ1	−2.824	19.056	44.700	0.911	0.005**
BZ3 - PZ2	−1.701	19.056	34.500	0.644	0.089
BZ3 - PZ3	−1.040	19.056	28.500	0.511	0.298
BZ3 - Z4	0.799	19.056	13.500	0.246	0.425
PZ1 - PZ2	0.990	44.700	34.500	0.600	0.322
PZ1 - PZ3	1.573	44.700	28.500	0.880	0.116
PZ1 - Z4	3.678	44.700	13.500	1.000	< .001***
PZ2 - PZ3	0.583	34.500	28.500	0.360	0.560
PZ2 - Z4	2.476	34.500	13.500	0.843	0.013*
PZ3 - Z4	1.768	28.500	13.500	0.800	0.077

* p < .05, ** p < .01, *** p < .001

Note. Rank-biserial correlation based on individual Mann-Whitney tests.

Supplementary Table 18. Kruskal-Wallis test statistics for non-activated microglia numbers in the DL of different zones. Results used in Figure 6D.

Factor	Statistic	df	p
Zone	33.111	6	< .001

Supplementary Table 19. Dunn's post hoc comparisions for non-activated microglia numbers in the DL of different zones. Results used in Figure 6D.

Comparison	z	W_i	W_j	r_{rb}	p
BZ1 - BZ2	0.788	15.889	9.833	0.160	0.431
BZ1 - BZ3	−1.692	15.889	28.889	0.531	0.091
BZ1 - PZ1	−0.870	15.889	23.800	0.467	0.384
BZ1 - PZ2	−2.014	15.889	34.200	0.622	0.044*
BZ1 - PZ3	−3.213	15.889	45.100	0.911	0.001**
BZ1 - Z4	−3.759	15.889	42.071	0.849	< .001***
BZ2 - BZ3	−2.480	9.833	28.889	0.802	0.013*
BZ2 - PZ1	−1.536	9.833	23.800	0.933	0.125
BZ2 - PZ2	−2.680	9.833	34.200	1.000	0.007**
BZ2 - PZ3	−3.879	9.833	45.100	1.000	< .001***
BZ2 - Z4	−4.629	9.833	42.071	1.000	< .001***
BZ3 - PZ1	0.560	28.889	23.800	0.333	0.576
BZ3 - PZ2	−0.584	28.889	34.200	0.222	0.559
BZ3 - PZ3	−1.783	28.889	45.100	0.689	0.075
BZ3 - Z4	−1.893	28.889	42.071	0.595	0.058
PZ1 - PZ2	−1.009	23.800	34.200	0.480	0.313
PZ1 - PZ3	−2.066	23.800	45.100	1.000	0.039*
PZ1 - Z4	−2.152	23.800	42.071	0.829	0.031*
PZ2 - PZ3	−1.057	34.200	45.100	0.520	0.290
PZ2 - Z4	−0.927	34.200	42.071	0.357	0.354
PZ3 - Z4	0.357	45.100	42.071	0.157	0.721

* p < .05, ** p < .01, *** p < .001

Note. Rank-biserial correlation based on individual Mann-Whitney tests.

Supplementary Table 20. MOTIQ all morphometric data for microglia in SRH induced Z1. Dataset used in Figure 7.

Number	Image	particle nr	x center [micron]	y center [micron]	x center of mass [mic	y center of mass [mic
**Cell1**	MAX_C2-2	1	630,0255	577,7293	629,0373	579,5862
**Cell2**	MAX_C2-2	1	715,4603	171,6088	715,7786	171,9788
**Cell3**	MAX_C2-2	1	512,1269	438,5721	512,7374	438,9288
**Cell4**	MAX_C2-2	1	136,025	366,8211	139,621	368,1278
**Cell5**	MAX_C2-2	1	680,1264	640,9854	679,7391	640,8443
**Cell6**	MAX_C2-2	1	768,1615	172,6144	768,3962	172,0394
**Cell7**	MAX_C2-2	1	56,18495	817,6335	55,35903	816,5622
**Cell8**	MAX_C2-2	1	179,9786	813,5516	180,7596	813,4594
**Cell9**	MAX_C2-2	1	321,3559	747,9677	322,0398	749,0344
**Cell10**	MAX_C2-2	1	199,4675	528,9584	199,6074	528,258
**Cell11**	MAX_C2-2	1	744,9459	39,10442	743,2532	37,2877
**Cell12**	MAX_C2-2	1	92,76342	459,5602	91,48284	459,6267
**Cell13**	MAX_C2-2	1	286,0557	198,0103	285,4501	195,4623
**Cell14**	MAX_C2-2	1	220,4994	564,0994	219,5736	565,0703
**Cell15**	MAX_C2-2	1	623,0887	206,3748	623,7762	204,1172

x span [micron]	y span [micron]	average intensity	minimum intensity	maximum intensity	standard d
84,41037	77,49149	61,071	13	255	49,19314
28,36742	48,43218	184,3678	29	255	84,09811
69,88072	62,96184	75,71261	18	255	48,11387
60,19428	48,43218	149,7941	28	255	85,95036
62,26995	66,42128	97,66032	22	255	75,88064
24,90798	31,82686	96,25026	27	255	46,61964
53,2754	65,72939	124,1289	27	255	77,90384
42,89708	38,74575	193,224	27	255	76,55544
69,18883	74,72394	100,5979	26	255	72,03033
36,67008	29,05931	190,7167	35	255	78,13253
45,66463	47,74029	162,1288	33	255	80,56582
77,49149	99,63192	87,19036	21	255	64,32722
70,57261	74,72394	85,96769	24	255	60,11129
62,96184	65,0375	70,95761	18	255	57,8327
47,74029	80,25904	78,16733	21	255	61,85532

eviation of area [micron^2]	outline [micron]	ramification index	spanned area (conve:	spanned outline (con
1368,63	1675,753	12,77798	4373,489	321,0362
727,6383	332,1064	3,473078	1075,66	149,4479
1510,807	1025,378	7,441745	3139,855	261,5338
1094,808	734,7854	6,264501	1795,639	214,4854
1106,297	934,0492	7,921891	3009,646	253,2311
453,3378	168,8207	2,236711	488,7623	109,3184
1344,695	756,9258	5,822862	2316,475	235,242
833,4331	283,6742	2,771913	1101,989	159,1343
1630,963	1574,738	10,99971	4071,424	285,058
542,3778	243,5447	2,950005	873,166	128,6912
858,326	444,1923	4,277007	1281,505	184,0423
1370,545	1466,803	11,17687	4648,268	351,4793
1229,805	1345,031	10,81955	3209,268	286,4418
1163,264	1398,998	11,57106	3404,581	251,8473
964,1208	1255,085	11,40257	3040,284	251,8473

spanned area center :	spanned area center ;	polarity vector x (bina	polarity vector y (bina	polarity vector length	polarity inc
628,7111	575,4946	-1,31444	-2,23467	2,592585	0,034743
714,3531	172,6369	-1,10718	1,028126	1,510925	0,040827
511,0327	439,4456	-1,09419	0,873523	1,400104	0,022144
133,7212	365,8175	-2,30383	-1,00352	2,512898	0,052555
681,3713	639,7158	1,244908	-1,26964	1,778138	0,028725
767,7964	172,4222	-0,36505	-0,19229	0,412599	0,01654
55,17455	816,7618	-1,0104	-0,87168	1,334437	0,024571
178,215	812,8581	-1,76355	-0,69341	1,894971	0,050589
322,2959	747,4335	0,94001	-0,5342	1,081197	0,015017
197,4912	530,6594	-1,97624	1,700984	2,607466	0,078202
746,5334	40,10975	1,587522	1,005326	1,879071	0,046519
93,54066	459,0447	0,777242	-0,5155	0,932656	0,012123
288,8747	202,735	2,819035	4,724766	5,501852	0,08607
222,3683	563,0562	1,868955	-1,04317	2,140373	0,032509
621,132	209,954	-1,95674	3,579248	4,079196	0,065564

dex (binary	polarity vector x [mic	polarity vector y [mic	polarity vector length	polarity index	ID of largest skeleton
	-0,32617	-4,09155	4,104534	0,055004	1
	-1,42544	0,658099	1,570023	0,042424	1
	-1,70471	0,516814	1,781327	0,028173	1
	-5,8998	-2,31028	6,336013	0,132511	1
	1,632139	-1,12848	1,984274	0,032054	1
	-0,59974	0,382804	0,711499	0,028521	1
	-0,18447	0,199665	0,271838	0,005005	1
	-2,54457	-0,60122	2,614628	0,069802	1
	0,256114	-1,60089	1,621248	0,022518	1
	-2,11617	2,401394	3,200762	0,095995	2
	3,280152	2,822051	4,327051	0,107122	1
	2,057826	-0,58202	2,138551	0,027798	1
	3,424648	7,272777	8,038749	0,125757	1
	2,794754	-2,0141	3,444885	0,052322	2
	-2,64427	5,836819	6,407854	0,102991	2

# found skls	# branches (largest sl	# junctions (largest sl	# tips (largest skl)	# triple points (larges	# quadrupl
2	41	22	14	20	2
1	4	2	2	2	0
3	21	10	11	9	1
1	19	10	6	8	2
1	13	6	7	5	1
1	1	0	2	0	0
1	10	4	7	3	1
1	3	1	3	1	0
1	36	17	15	13	3
2	3	1	3	1	0
1	13	7	5	7	0
4	15	7	9	7	0
2	42	24	9	21	3
7	19	9	11	9	0
6	22	10	10	8	1

e points (la	# junction voxels (larg	# slab voxels (largest	tree length (largest skl) [mic	average branch lengt	maximum branch len
42	386	344,319856	8,398045	33,64474	
6	114	102,919982	25,73	47,86744	
23	289	259,549466	12,3595	34,55368	
22	203	192,477035	10,13037	32,02355	
15	219	194,873332	14,99026	30,11576	
0	9	8,63842	8,63842	8,63842	
11	208	183,849887	18,38499	39,51106	
3	85	71,154222	23,71807	36,69888	
39	424	388,107508	10,78076	32,65983	
3	45	43,47869	14,4929	27,87222	
9	150	134,202469	10,32327	20,28181	
13	313	257,199143	17,14661	53,35337	
53	362	328,574305	7,823198	27,75351	
21	276	243,2223	12,80117	24,88761	
26	279	251,043691	11,41108	28,80152	

shortest path (largest	# branches (all skls)	# junctions (all skls)	# tips (all skls)	# triple points (all skl	# quadrupl
126,178	41	22	14	20	2
65,83617	4	2	2	2	0
99,83704	21	10	11	9	1
61,27499	19	10	6	8	2
88,24282	13	6	7	5	1
8,63842	1	0	2	0	0
88,1891	10	4	7	3	1
63,37557	3	1	3	1	0
105,8266	36	17	15	13	3
35,81875	3	1	3	1	0
56,91116	13	7	5	7	0
135,8644	15	7	9	7	0
132,8837	42	24	9	21	3
95,80442	18	9	9	9	0
93,32345	21	10	8	8	1

le points (a	# junction voxels (all s	# slab voxels (all skls	tree length (all skls) [	average branch length	maximum branch len
42	387	346,2768	8,244686	33,64474	
6	114	102,92	25,73	47,86744	
23	289	260,2414	11,82915	34,55368	
22	203	192,477	10,13037	32,02355	
15	219	194,8733	14,99026	30,11576	
0	9	8,63842	8,63842	8,63842	
11	208	183,8499	18,38499	39,51106	
3	85	71,15422	23,71807	36,69888	
39	424	388,1075	10,78076	32,65983	
3	54	51,25734	12,81434	27,87222	
9	150	134,2025	10,32327	20,28181	
13	320	265,551	14,75283	53,35337	
53	365	332,2016	7,725619	27,75351	
21	278	247,6602	10,76784	24,88761	
26	283	258,0117	9,923529	28,80152	

shortest path (all skl) [micron]
128,1349
65,83617
100,5289
61,27499
88,24282
8,63842
88,1891
63,37557
105,8266
43,5974
56,91116
144,2162
136,511
100,2423
100,2915

Supplementary Table 21. MOTIQ all morphometric data for microglia in SRH induced Z2. Dataset used in Figur

Number	Image	particle nr	x center [micron]	y center [micron]	x center of mass [mic	y center of
**Cell1**	MAX_C2-2(	1	146,5574	137,8573	145,8872	138,5862
**Cell2**	MAX_C2-2(	1	186,8595	340,7834	186,9845	340,309
**Cell3**	MAX_C2-2(	1	160,1245	424,5538	160,2764	425,4196
**Cell4**	MAX_C2-2(	1	321,1526	719,1537	319,9605	717,0357
**Cell5**	MAX_C2-2(	1	635,2519	738,8677	633,9826	740,6835
**Cell6**	MAX_C2-2(	1	575,3874	675,5	571,9903	675,6202
**Cell7**	MAX_C2-2(	1	481,9881	529,1184	481,8535	526,9606
**Cell8**	MAX_C2-2(	1	349,1853	493,5751	349,0838	491,8976
**Cell9**	MAX_C2-2(	1	648,9232	462,4643	648,5981	459,6273
**Cell10**	MAX_C2-2(	1	579,5538	354,9546	579,3557	354,0641
**Cell11**	MAX_C2-2(	1	582,8863	247,8594	582,373	246,3401
**Cell12**	MAX_C2-2(	1	447,889	606,0071	448,1421	606,3552
**Cell13**	MAX_C2-2(	1	444,4617	674,3089	445,1501	672,5397
**Cell14**	MAX_C2-2(	1	561,274	875,5477	563,164	874,5572
**Cell15**	MAX_C2-2(	1	640,5775	136,1889	640,2489	136,1857

e 7.

mass [mic x span [micron]	y span [micron]	average intensity	minimum intensity	maximum
89,25359	93,40492	48,07103	7	255
89,94548	69,18883	69,1727	5	255
58,81051	78,87527	90,1225	7	255
65,0375	42,20519	98,98145	7	255
23,5242	31,13497	95,85924	7	255
49,12407	51,19973	75,22971	7	255
106,5508	78,87527	73,46379	5	255
91,32926	67,80505	79,53042	7	255
62,96184	91,32926	70,548	7	255
81,64282	91,32926	58,12976	5	255
98,94003	123,848	54,36746	5	255
80,95093	74,03205	69,87626	7	255
40,82141	65,72939	66,94786	7	255
92,71303	62,96184	56,93618	5	255
71,2645	106,5508	63,85362	5	255

intensity	standard deviation of	area [micron^2]	outline [micron]	ramification index	spanned a
	50,33986	1718,567	1923,449	13,08858	5846,957
	72,55273	2076,163	1887,471	11,68543	4744,01
	86,39692	1410,757	1165,14	8,750786	3350,009
	93,16411	1109,648	830,266	7,03104	2034,515
	91,14466	326,4798	261,5338	4,083144	522,7507
	70,87854	696,0435	568,7322	6,081136	1694,153
	72,62156	1672,132	1299,366	8,963784	4730,128
	76,19128	1447,617	1224,642	9,079824	3810,527
	69,00506	1780,32	1681,289	11,24057	4114,029
	59,43487	2062,28	1906,844	11,84504	5683,238
	53,70888	2015,367	1938,671	12,1821	7038,943
	68,67164	1419,852	1356,101	10,15233	4017,808
	71,37557	826,2525	799,8229	7,849329	1819,575
	62,52102	1245,123	1368,555	10,94085	3588,885
	65,79131	1671,175	1675,753	11,56363	4723,426

rea (conve:	spanned outline (con	spanned area center :	spanned area center y	polarity vector x (bina	polarity ve
	361,1657	146,4776	140,0378	-0,07972	2,180463
	314,1173	188,4182	340,4086	1,558684	-0,37483
	272,604	159,8749	421,8964	-0,24954	-2,65733
	211,7178	322,0063	722,1956	0,8537	3,041899
	106,5508	635,1135	737,0923	-0,13832	-1,77542
	197,8801	579,2044	672,9755	3,817003	-2,52442
	368,0846	481,3012	533,5829	-0,68692	4,464589
	314,1173	348,5403	492,9343	-0,64502	-0,64088
	305,8146	649,8283	462,1295	0,905043	-0,3348
	343,1766	580,0962	354,7337	0,542402	-0,22093
	441,4247	581,8549	252,0069	-1,0314	4,147486
	307,1984	447,4109	604,9262	-0,47808	-1,08099
	210,334	444,0752	676,6476	-0,38651	2,338668
	308,5822	558,8468	878,2439	-2,42715	2,696274
	352,863	641,1933	135,927	0,615856	-0,26198

ctor y (bina	polarity vector length	polarity index (binary	polarity vector x [mic	polarity vector y [mic	polarity ve
	2,18192	0,025288	0,590447	1,451581	1,567072
	1,60312	0,020627	1,433752	0,09953	1,437202
	2,669017	0,040867	-0,40149	-3,52312	3,54592
	3,159423	0,062076	2,045817	5,159884	5,550655
	1,780796	0,069026	1,130957	-3,59116	3,765037
	4,576267	0,098533	7,214183	-2,64469	7,683671
	4,517124	0,058206	-0,55229	6,622386	6,645376
	0,909271	0,013054	-0,54351	1,036685	1,170523
	0,964985	0,013333	1,230188	2,50215	2,78821
	0,585669	0,006885	0,74048	0,669551	0,998303
	4,273808	0,045145	-0,51811	5,666794	5,69043
	1,181987	0,016526	-0,7312	-1,42907	1,605271
	2,370393	0,049247	-1,07496	4,107828	4,24615
	3,627802	0,053667	-4,31721	3,686771	5,677199
	0,669261	0,00863	0,944403	-0,25872	0,979199

ctor length polarity index	ID of largest skeleton	# found skls	# branches (largest sl	# junctions
0,018162	1	6	27	13
0,018492	1	2	59	32
0,054294	1	2	23	11
0,109058	1	1	13	7
0,145938	1	1	1	0
0,165439	1	4	7	3
0,08563	1	3	21	10
0,016805	1	1	27	13
0,038524	1	2	48	27
0,011736	1	4	34	18
0,060109	1	4	48	25
0,022444	1	4	29	14
0,088218	1	2	24	13
0,083985	1	1	24	11
0,012627	1	3	33	18

s (largest sl	# tips (largest skl)	# triple points (larges	# quadruple points (la	# junction voxels (larg	# slab voxe
	14	12	1	28	421
	17	28	3	53	580
	12	10	1	19	300
	4	6	1	13	234
	2	0	0	0	42
	5	3	0	9	116
	12	10	0	18	353
	14	12	1	28	326
	13	25	2	57	481
	12	16	2	34	478
	20	24	1	50	585
	15	13	1	27	366
	8	12	1	21	237
	14	10	1	20	367
	11	17	1	35	454

els (largest	tree length (largest sk	average branch lengt	maximum branch len	shortest path (largest	# branches
	370,7388	13,73107	39,72811	137,0396	27
	517,0391	8,763374	27,08199	133,5665	59
	266,8041	11,60018	30,63977	122,6982	23
	201,5615	15,50473	29,08811	95,567	13
	35,76958	35,76958	35,76958	35,76958	1
	97,90045	13,98578	21,83347	72,08353	7
	298,029	14,19186	29,03894	141,4283	21
	287,7445	10,6572	41,01809	118,1619	27
	429,7301	8,952711	26,20185	121,7688	48
	412,7111	12,13856	34,97935	128,3723	34
	511,2135	10,65028	32,42885	183,3896	48
	315,1334	10,86667	26,67669	127,7296	29
	208,9348	8,705618	23,50384	75,87873	24
	319,244	13,30183	34,62322	110,7145	24
	394,3121	11,94885	44,76413	140,5685	33

s (all skls)	# junctions (all skls)	# tips (all skls)	# triple points (all skl	# quadruple points (a	# junction
	13	14	12	1	28
	32	17	28	3	53
	11	12	10	1	19
	7	4	6	1	13
	0	2	0	0	0
	3	5	3	0	9
	10	12	10	0	18
	13	14	12	1	28
	27	13	25	2	57
	18	12	16	2	34
	25	20	24	1	50
	14	15	13	1	27
	13	8	12	1	21
	11	14	10	1	20
	18	11	17	1	35

voxels (all :	# slab voxels (all skls	tree length (all skls) [	average branch lengtl	maximum branch len	shortest pa
	469	410,5613	12,83004	39,72811	176,8621
	583	520,3798	8,672997	27,08199	136,9072
	301	268,4745	11,18644	30,63977	124,3685
	234	201,5615	15,50473	29,08811	95,567
	42	35,76958	35,76958	35,76958	35,76958
	119	101,9331	11,3259	21,83347	76,11615
	355	300,1047	13,64112	29,03894	143,504
	326	287,7445	10,6572	41,01809	118,1619
	486	434,4546	8,866421	26,20185	126,4934
	485	418,9381	11,63717	34,97935	134,5993
	588	515,938	10,11643	32,42885	188,1142
	371	320,9551	10,02985	26,67669	133,5513
	237	209,6267	8,385069	23,50384	76,57062
	367	319,244	13,30183	34,62322	110,7145
	457	397,6529	11,69567	44,76413	143,9093

ath (all skl) [micron]

Supplementary Table 22. MOTIQ all morphometric data for microglia in SRH induced Z3. Dataset used in Figur

Number	Image	particle nr	x center [micron]	y center [micron]	x center of mass [mic	y center of
**Cell1**	MAX_C2-2	1	100,6086	121,4463	99,24953	121,995
**Cell2**	MAX_C2-2	1	79,43455	238,7201	78,79834	240,0548
**Cell3**	MAX_C2-2	1	350,909	153,1369	350,0536	154,7375
**Cell4**	MAX_C2-2	1	159,5279	434,7764	159,8564	432,7214
**Cell5**	MAX_C2-2	1	628,3208	477,4907	626,7235	476,6166
**Cell6**	MAX_C2-2	1	351,416	152,696	350,3036	154,4368
**Cell7**	MAX_C2-2	1	338,3787	464,483	338,5943	464,2747
**Cell8**	MAX_C2-2	1	445,0178	503,3579	443,8158	504,4899
**Cell9**	MAX_C2-2	1	492,8015	671,4658	494,6478	670,4149
**Cell10**	MAX_C2-2	1	213,0048	96,61634	213,2826	94,66971
**Cell11**	MAX_C2-2	1	462,2742	314,0182	464,377	314,6652
**Cell12**	MAX_C2-2	1	254,6529	166,3169	255,5475	163,3766
**Cell13**	MAX_C2-2	1	250,9985	513,539	249,9955	515,4767
**Cell14**	MAX_C2-2	1	169,2103	560,8308	167,3965	558,408
**Cell15**	MAX_C2-2	1	111,5028	641,3097	112,0511	642,0051

e 7.

mass [mic x span [micron]	y span [micron]	average intensity	minimum intensity	maximum
83,0266	105,8589	55,0255	8	255
80,95093	91,32926	60,08341	8	255
90,63737	115,5453	69,32181	8	255
114,8535	114,8535	48,27503	8	255
109,3184	94,7887	67,64943	10	255
92,71303	112,0859	68,93107	8	255
87,17793	113,4697	57,98191	8	255
85,79415	91,32926	63,14736	8	255
92,71303	121,0805	54,91832	10	255
80,25904	71,95638	92,8888	8	255
96,17247	111,394	54,66155	8	255
73,34016	98,94003	64,97207	10	255
105,8589	83,0266	68,30877	8	255
99,63192	98,94003	59,41212	8	255
88,5617	120,3886	72,72669	13	255

intensity	standard deviation	of area [micron^2]	outline [micron]	ramification index	spanned a
	60,99358	1783,193	2310,907	15,43755	5911,583
	61,76496	1589,794	1837,655	13,00136	4799,541
	71,98116	1828,191	2220,961	14,65296	6761,292
	51,68288	2175,734	3013,865	18,22703	9174,945
	64,91988	2453,864	2803,531	15,96523	7105,484
	71,97171	1833,457	2245,869	14,796	6783,313
	60,40002	1798,99	2204,356	14,66096	6478,853
	61,6153	1731,492	1880,552	12,74883	5471,17
	54,67359	1687,929	2333,047	16,01922	6004,452
	88,52634	1231,241	1279,993	10,29038	4000,096
	59,41094	2398,813	2824,288	16,26694	7366,859
	63,22974	1268,101	1475,106	11,68534	3951,268
	69,01262	1747,289	1962,195	13,24204	5241,868
	61,23554	1982,814	2352,42	14,90284	6829,269
	65,74839	2238,445	2709,435	16,15476	7382,178

rea (conve	spanned outline (con	spanned area center	spanned area center	polarity vector x (bina	polarity ve
	375,0035	101,5624	122,2124	0,953745	0,766141
	340,409	82,44223	237,9894	3,007677	-0,73069
	408,2141	352,04	156,9106	1,130984	3,77361
	455,2625	159,4458	436,3843	-0,08209	1,607826
	404,0628	628,2966	475,4614	-0,02428	-2,02922
	405,4465	352,8416	155,3904	1,425617	2,694443
	398,5277	340,183	469,0258	1,804362	4,542829
	351,4793	446,5988	502,8329	1,580972	-0,52503
	424,8194	494,5644	672,1712	1,762946	0,705372
	301,6633	207,0857	101,1522	-5,91901	4,535895
	410,9817	465,6883	318,38	3,414087	4,361786
	340,409	256,1874	166,914	1,534463	0,597125
	373,6197	248,4096	514,4423	-2,58893	0,903246
	392,9926	172,0371	563,1495	2,826773	2,318692
	413,7492	110,3294	643,677	-1,17342	2,367249

ctor y (bina	polarity vector length	polarity index (binary	polarity vector x [mic	polarity vector y [mic	polarity ve
	1,223357	0,014101	2,312855	0,217443	2,323054
	3,095161	0,039594	3,643886	-2,06542	4,188539
	3,939449	0,042459	1,986434	2,173021	2,944137
	1,60992	0,014895	-0,4106	3,662914	3,685856
	2,029367	0,021336	1,573046	-1,15517	1,951638
	3,048345	0,032801	2,53806	0,95356	2,711277
	4,888049	0,053818	1,588725	4,751043	5,009637
	1,66587	0,019959	2,782993	-1,65696	3,238916
	1,898823	0,021717	-0,08342	1,756299	1,758279
	7,457144	0,104492	-6,19682	6,482523	8,967922
	5,539058	0,057193	1,311286	3,714837	3,939478
	1,646552	0,023214	0,639821	3,537369	3,594767
	2,741968	0,033563	-1,58589	-1,03446	1,89345
	3,656088	0,039208	4,640549	4,741465	6,63447
	2,642116	0,027252	-1,72177	1,671836	2,399899

ctor length polarity index	ID of largest skeleton	# found skls	# branches (largest sl	# junctions
0,026776	1	7	50	26
0,053581	1	4	41	21
0,031731	1	3	57	31
0,034102	2	11	64	34
0,020519	1	5	72	40
0,029174	1	3	59	32
0,055157	1	3	61	33
0,038807	1	2	40	20
0,020109	3	4	56	28
0,125661	3	4	27	14
0,040676	2	6	55	30
0,050681	1	5	31	16
0,023177	1	3	47	25
0,071148	1	2	62	32
0,024754	2	4	61	33

s (largest sl	# tips (largest skl)	# triple points (larges	# quadruple points (la	# junction voxels (larg	# slab voxe
	19	23	3	52	510
	14	17	3	50	471
	19	29	2	71	603
	25	33	1	71	676
	23	39	1	76	729
	20	30	2	74	603
	19	29	4	63	626
	20	20	0	34	477
	24	25	2	47	555
	12	14	0	28	315
	15	25	5	70	651
	14	16	0	34	349
	18	24	1	41	532
	24	28	4	66	630
	21	31	2	66	675

els (largest	tree length (largest sk	average branch lengtl	maximum branch len	shortest path (largest	# branches
	453,5409	9,070818	18,25532	140,0445	50
	417,574	10,18473	25,12503	124,1515	41
	538,1722	9,441618	32,04391	152,2119	57
	598,3795	9,34968	43,83482	168,322	64
	649,0637	9,014773	34,09921	149,3748	72
	540,8211	9,166459	32,04391	139,9954	59
	556,7633	9,127267	35,72041	155,5035	61
	408,6005	10,21501	35,72041	143,7414	40
	489,4878	8,740853	27,70434	162,4715	56
	279,9704	10,36927	58,24576	123,9141	27
	582,4321	10,58967	27,36858	135,0826	55
	305,5702	9,857104	26,96328	137,9892	31
	462,3139	9,836465	32,14226	134,3415	47
	570,0522	9,19439	25,34208	155,5678	62
	593,1802	9,724265	49,32075	158,0828	61

s (all skls)	# junctions (all skls)	# tips (all skls)	# triple points (all skl	# quadruple points (a	# junction
	26	19	23	3	52
	21	14	17	3	50
	31	19	29	2	71
	34	25	33	1	71
	40	23	39	1	76
	32	20	30	2	74
	33	19	29	4	63
	20	20	20	0	34
	28	24	25	2	47
	14	12	14	0	28
	30	15	25	5	70
	16	14	16	0	34
	25	18	24	1	41
	32	24	28	4	66
	33	21	31	2	66

voxels (all s	# slab voxels (all skls	tree length (all skls) [	average branch length	maximum branch len	shortest pa
	540	483,7754	8,795916	18,25532	170,279
	475	423,5636	9,626445	25,12503	130,141
	606	541,9183	9,185055	32,04391	155,958
	737	652,4248	9,061455	43,83482	222,3673
	737	656,5557	8,872375	34,09921	156,8669
	608	546,2375	8,954713	32,04391	145,4118
	635	566,3802	8,990162	35,72041	165,1204
	481	412,3465	10,05723	35,72041	147,4874
	587	516,8563	8,760277	27,70434	189,8401
	331	295,1224	9,837412	58,24576	139,0661
	672	603,8602	10,06434	27,36858	156,5108
	349	307,5272	9,319005	26,96328	139,9462
	538	469,8551	9,58888	32,14226	141,8828
	656	591,8856	9,39501	25,34208	177,4013
	683	601,9373	9,40527	49,32075	166,8399

ath (all skl) [micron]

Supplementary Table 23. MOTIQ all morphometric data for microglia in Z4. Dataset used in Figure 7.

Number	Image	particle nr	x center [micron]	y center [micron]	x center of mass [mic	y center of
Cell1	C2-MAX_2(	1	75,11582	268,0257	76,0671	267,0401
Cell2	C2-MAX_2(	1	141,7756	328,3206	141,6805	327,991
Cell3	C2-MAX_2(	1	84,0913	638,8578	84,69334	636,8514
Cell4	C2-MAX_2(	1	233,3271	218,6699	233,7374	217,566
Cell5	C2-MAX_2(	1	386,7752	96,52705	386,4025	96,73813
Cell6	C2-MAX_2(	1	519,8189	215,9131	520,6757	214,4183
Cell7	C2-MAX_2(	1	353,2634	577,7969	352,9515	577,3397
Cell8	C2-MAX_2(	1	506,9738	376,3097	505,9734	375,0787
Cell9	C2-MAX_2(	1	589,3846	460,4092	589,4851	460,352
Cell10	C2-MAX_2(	1	278,886	81,63011	279,102	81,67331
Cell11	C2-MAX_2(	1	453,6326	458,0788	454,1322	456,1161
Cell12	C2-MAX_2(	1	608,4803	178,0327	608,4542	178,231
Cell13	C2-MAX_2(	1	617,2353	395,0897	617,3485	395,2668
Cell14	C2-MAX_2(	1	305,77	356,9396	305,0681	354,1987
Cell15	C2-MAX_2(	1	88,59212	181,2289	86,9226	181,2788

mass [mic x span [micron]	y span [micron]	average intensity	minimum intensity	maximum
98,94003	72,64827	56,76285	27	255
95,48059	121,0805	54,41978	28	255
110,7021	143,9128	49,11016	25	255
67,80505	75,41583	60,48834	30	255
143,2209	115,5453	55,51757	28	255
115,5453	109,3184	55,80297	29	255
73,34016	83,0266	58,96556	29	255
93,40492	124,5399	58,46823	29	255
105,167	76,7996	54,32922	29	255
116,2372	92,71303	56,04736	28	255
96,17247	133,5344	53,34617	28	255
93,40492	93,40492	49,73632	29	255
140,4533	115,5453	49,99565	28	255
75,41583	76,10771	51,56867	29	255
77,49149	83,0266	50,07531	25	255

intensity	standard deviation of	area [micron^2]	outline [micron]	ramification index	spanned a
	41,68257	1499,797	2032,768	14,80699	4638,216
	38,50388	2264,774	3309,994	19,62049	7550,684
	34,00444	2798,535	3887,028	20,72752	10441,13
	42,20488	1210,656	1721,418	13,95632	3927,811
	38,23943	3147,514	4554,009	22,8984	10490,44
	36,82143	2801,408	3900,866	20,79065	8534,432
	42,92671	1821,011	2360,723	15,60572	4481,199
	39,44204	2681,73	3524,479	19,19917	7261,065
	34,75357	1784,15	2522,625	16,84737	5887,168
	38,74573	2436,152	3491,268	19,95382	8439,647
	34,1633	3393,571	4206,681	20,37071	8689,055
	31,53402	1653,941	2644,397	18,34264	5947,007
	31,63237	3299,744	4898,569	24,05604	10791,55
	35,92859	1387,3	2097,805	15,88821	4346,203
	37,22808	1481,127	2136,551	15,66074	4578,856

rea (conve:	spanned outline (con	spanned area center :	spanned area center ;	polarity vector x (bina	polarity ve
340,409	74,81616	268,272	-0,29965	0,246361	
430,3545	141,198	328,7941	-0,5776	0,473494	
506,4622	85,13308	641,564	1,041784	2,706241	
283,6742	232,1408	218,1567	-1,18634	-0,51326	
513,3811	391,3862	96,37173	4,611032	-0,15532	
446,9598	521,8675	214,9668	2,048581	-0,94633	
309,966	353,1736	578,252	-0,08971	0,455047	
431,7383	508,7621	376,4318	1,788295	0,122083	
359,7819	589,6832	461,8588	0,298617	1,449693	
415,133	277,6544	81,5814	-1,2316	-0,04871	
456,6463	454,5472	460,9081	0,914525	2,82925	
370,8521	610,0055	178,8591	1,525152	0,826468	
507,846	620,8438	396,1655	3,608511	1,075822	
300,2795	307,5024	359,0752	1,732376	2,13557	
318,2686	87,23236	180,51	-1,35976	-0,71885	

ctor y (bina	polarity vector length	polarity index (binary	polarity vector x [mic	polarity vector y [mic	polarity ve
	0,387925	0,005048	-1,25094	1,231928	1,755703
	0,746872	0,007617	-0,48252	0,803078	0,936889
	2,899837	0,02515	0,439741	4,712613	4,733085
	1,292605	0,018278	-1,59669	0,590634	1,702429
	4,613647	0,03992	4,983745	-0,3664	4,997196
	2,256597	0,021648	1,191801	0,548494	1,311959
	0,463806	0,00614	0,222101	0,912297	0,938944
	1,792457	0,018642	2,788655	1,353019	3,099557
	1,480129	0,017096	0,198139	1,50682	1,519792
	1,23256	0,01189	-1,44758	-0,09191	1,450496
	2,973384	0,028269	0,414912	4,791994	4,809923
	1,734687	0,019935	1,551246	0,628118	1,673588
	3,765467	0,032123	3,495299	0,898628	3,608968
	2,74987	0,036966	2,43423	4,876531	5,450324
	1,538079	0,020144	0,309763	-0,76877	0,82883

ctor length polarity index	ID of largest skeleton	# found skls	# branches (largest sl	# junctions
0,022847	2	3	64	37
0,009555	1	2	135	79
0,04105	1	4	112	66
0,024073	1	1	48	27
0,043239	1	2	132	76
0,012586	1	1	128	76
0,01243	1	1	99	58
0,032236	1	2	130	77
0,017554	1	5	74	41
0,013993	1	3	128	75
0,04573	1	1	143	87
0,019233	1	7	82	47
0,030788	1	3	180	108
0,073268	1	3	87	53
0,010855	1	5	62	35

s (largest sl	# tips (largest skl)	# triple points (larges	# quadruple points (la	# junction voxels (larg	# slab voxe
	15	35	2	68	614
	22	69	9	164	1052
	21	61	5	127	1109
	12	23	2	60	525
	29	69	7	156	1234
	21	69	7	151	1167
	9	45	11	140	780
	21	69	8	158	1068
	21	38	2	85	725
	26	72	1	145	1090
	18	80	7	183	1287
	20	44	3	86	774
	26	98	8	246	1424
	9	47	6	110	689
	16	32	3	64	544

els (largest	tree length (largest sk	average branch lengtl	maximum branch len	shortest path (largest	# branches
	551,8952	8,623362	33,81262	125,5352	63
	987,3998	7,314073	31,97438	170,062	135
	978,0478	8,73257	40,08879	187,9815	112
	469,3784	9,778716	30,13613	109,3352	48
	1111,015	8,41678	57,84046	174,1325	132
	1054,839	8,240933	30,47189	189,7948	128
	726,9479	7,342908	27,19615	131,7361	99
	996,816	7,667816	31,23332	162,4649	130
	645,031	8,716636	23,33596	142,7361	74
	992,4324	7,753378	42,63929	186,3111	128
	1171,951	8,195464	29,61212	161,5869	143
	671,6456	8,1908	21,30491	136,4822	82
	1348,022	7,489009	33,93133	192,2804	180
	631,8852	7,263048	20,73628	114,2972	87
	491,3166	7,924462	38,53713	108,8808	62

s (all skls)	# junctions (all skls)	# tips (all skls)	# triple points (all skl	# quadruple points (a	# junction
	37	13	35	2	68
	79	22	69	9	164
	66	21	61	5	127
	27	12	23	2	60
	76	29	69	7	156
	76	21	69	7	151
	58	9	45	11	140
	77	21	69	8	158
	41	21	38	2	85
	75	26	72	1	145
	87	18	80	7	183
	47	20	44	3	86
	108	26	98	8	246
	53	9	47	6	110
	35	16	32	3	64

voxels (all s	# slab voxels (all skls	tree length (all skls) [	average branch length	maximum branch len	shortest pa
	614	551,8952	8,623362	33,81262	125,5352
	1052	988,3783	7,267488	31,97438	171,0404
	1109	978,7397	8,661413	40,08879	188,6734
	525	469,3784	9,778716	30,13613	109,3352
	1234	1111,015	8,41678	57,84046	174,1325
	1167	1054,839	8,240933	30,47189	189,7948
	780	726,9479	7,342908	27,19615	131,7361
	1072	1000,562	7,637878	31,23332	166,2109
	728	651,3072	8,350092	23,33596	149,0123
	1095	996,8703	7,727677	42,63929	190,7491
	1287	1171,951	8,195464	29,61212	161,5869
	795	692,5006	7,959777	21,30491	157,3372
	1428	1352,746	7,432671	33,93133	197,0049
	692	634,9393	7,215219	20,73628	117,3513
	563	508,8309	7,828167	38,53713	126,395

ath (all skl) [micron]

Supplementary Table 24. MOTIQ all morphometric data for microglia in subretinally injected PBS Z1. Dataset u

Number	Image	particle nr	x center [micron]	y center [micron]	x center of mass [mic	y center of
**Cell1**	MAX_C1-2(	1	203,514	461,1402	203,9672	461,5314
**Cell2**	MAX_C1-2(	1	540,8729	505,7271	541,6266	506,5217
**Cell3**	MAX_C1-2(	1	279,6741	571,9048	279,5356	571,364
**Cell4**	MAX_C1-2(	1	445,3165	256,4463	445,0886	256,2979
**Cell5**	MAX_C1-2(	1	294,682	197,346	295,8604	195,6189
**Cell6**	MAX_C1-2(	1	268,0629	547,2035	268,5199	546,8617
**Cell7**	MAX_C1-2(	1	170,684	88,45469	168,7633	89,47454
**Cell8**	MAX_C1-2(	1	578,6947	307,0512	576,9119	307,3872
**Cell9**	MAX_C1-2(	1	518,5227	494,8412	517,2609	494,9851
**Cell10**	MAX_C1-2(	1	155,3223	219,0761	155,3089	219,8139
**Cell11**	MAX_C1-2(	1	248,2948	516,9424	247,4979	517,5714
**Cell12**	MAX_C1-2(	1	133,4534	238,4271	134,0362	238,568
**Cell13**	MAX_C1-2(	1	574,3861	222,2144	574,1444	222,0404
**Cell14**	MAX_C1-2(	1	114,8664	189,6106	113,4095	188,4637
**Cell15**	MAX_C1-2(	1	414,6409	462,3787	413,977	461,8145

ısed in Figure 7.

mass [mic	x span [micron]	y span [micron]	average intensity	minimum intensity	maximum
	84,41037	71,95638	107,1088	45	255
	79,56716	60,88617	116,6137	46	255
	65,72939	71,95638	78,23612	9	255
	77,49149	80,25904	71,10956	8	255
	82,33471	100,3238	96,76326	36	240
	81,64282	89,25359	91,58607	35	240
	83,71848	99,63192	85,08957	24	255
	63,65372	75,41583	92,15894	22	255
	56,73484	53,96729	149,1382	2	255
	123,848	36,67008	137,0557	2	255
	100,3238	68,49694	95,73947	30	255
	82,33471	80,25904	88,50807	28	255
	58,81051	80,95093	109,2609	36	255
	89,25359	114,8535	68,25973	22	255
	107,9346	76,7996	97,9711	29	255

intensity	standard deviation of	area [micron^2]	outline [micron]	ramification index	spanned ar
	59,1325	1571,124	2184,983	15,55027	4824,434
	65,98903	749,659	1140,232	11,7478	3154,695
	63,44935	2984,275	3891,18	20,09356	4094,402
	57,63693	2875,129	4149,946	21,83277	4317,002
	60,43404	1308,313	1819,666	14,19159	5271,069
	55,64157	1368,152	1857,028	14,16271	4108,284
	68,60638	1555,327	1653,613	11,82819	6161,469
	70,35986	1156,562	1003,238	8,321749	3164,748
	89,0888	772,637	604,7104	6,13698	1834,893
	89,60859	850,1879	723,7152	7,001729	2585,031
	69,98851	1488,308	1614,867	11,80825	4632,471
	65,97162	1660,164	1815,515	12,56954	4520,453
	74,31341	1157,998	1154,07	9,566943	3067,57
	54,28071	2386,845	2378,712	13,73488	7570,311
	70,93201	1938,294	1953,893	12,51947	6010,676

rea (conve›	spanned outline (con	spanned area center ›	spanned area center ›	polarity vector x (bina	polarity ve
308,5822	201,1808	463,18	-2,33319	2,039837	
278,1391	541,6105	506,3679	0,737622	0,6408	
271,2202	280,0103	572,2675	0,336154	0,362627	
312,7335	444,1433	256,8294	-1,17318	0,383015	
361,1657	293,6938	200,2454	-0,98818	2,89935	
339,0253	267,9935	543,803	-0,06937	-3,40051	
362,5495	173,5747	88,05976	2,890714	-0,39493	
273,9878	578,7762	308,7055	0,081493	1,654313	
218,6367	518,2656	496,099	-0,25712	1,257814	
316,8848	161,6934	218,7545	6,371098	-0,32169	
333,4902	252,4068	516,2684	4,112014	-0,67398	
322,4199	132,9819	238,9935	-0,47157	0,566339	
275,3715	571,0753	223,7967	-3,31084	1,582298	
405,4465	117,8963	194,4653	3,029933	4,854767	
366,7008	414,3834	462,8724	-0,25754	0,493727	

ctor y (bina	polarity vector length	polarity index (binary)	polarity vector x [micı	polarity vector y [micı	polarity ve
3,09915	0,039543	-2,78639	1,648641	3,237588
0,977093	0,015417	-0,01614	-0,15389	0,154739
0,494467	0,006848	0,474672	0,90351	1,02061
1,234121	0,016646	-0,94523	0,531476	1,084401
3,063126	0,03739	-2,16655	4,626476	5,108641
3,401221	0,047027	-0,52638	-3,05866	3,103624
2,917567	0,03294	4,811357	-1,41478	5,015052
1,656319	0,026093	1,864265	1,318367	2,283325
1,283824	0,026561	1,004667	1,113916	1,500055
6,379215	0,111193	6,384475	-1,05943	6,471778
4,166882	0,054256	4,908834	-1,30298	5,078819
0,736963	0,009714	-1,05431	0,425503	1,136939
3,669509	0,058716	-3,06908	1,756359	3,536102
5,722697	0,058289	4,486766	6,001635	7,493377
0,556859	0,006365	0,406406	1,057846	1,133227

ctor length	polarity index	ID of largest skeleton	# found skls	# branches (largest sl	# junctions
	0,041309	1	3	34	17
	0,002442	1	4	17	8
	0,014135	1	1	1	0
	0,014627	1	1	18	11
	0,062359	1	7	29	15
	0,042912	2	6	34	19
	0,056621	1	6	24	12
	0,03597	2	3	12	5
	0,031035	1	1	19	9
	0,112807	1	1	7	3
	0,06613	1	5	29	15
	0,014986	1	4	40	23
	0,056581	1	1	26	14
	0,076325	1	2	47	24
	0,012954	2	11	45	24

s (largest sl	# tips (largest skl)	# triple points (larges	# quadruple points (la	# junction voxels (larg	# slab voxe
	14	14	3	36	443
	10	8	0	14	225
	2	0	0	0	55
	2	8	2	30	354
	13	13	1	37	368
	11	19	0	37	388
	12	12	0	30	388
	8	4	1	14	247
	9	7	2	13	233
	5	3	0	7	225
	12	14	1	36	379
	11	23	0	43	424
	10	14	0	26	287
	20	22	2	52	644
	17	21	2	51	468

els (largest	tree length (largest sk	average branch lengtl	maximum branch len	shortest path (largest	# branches
	394,834	11,61277	31,45037	109,671	34
	197,4797	11,61645	30,82802	97,83091	17
	49,63615	49,63615	49,63615	49,63615	1
	319,5436	17,75242	59,37175	83,82526	18
	326,8593	11,27101	39,22902	143,5531	29
	347,0021	10,20594	36,64971	115,5826	34
	342,4166	14,26736	27,41775	143,9093	24
	201,8396	16,81997	34,74193	94,50411	11
	204,2103	10,74791	43,78565	94,58852	19
	182,7042	26,1006	64,59147	154,6437	7
	330,8094	11,40722	21,66559	133,2647	29
	387,4758	9,686894	28,80152	116,5899	40
	251,001	9,653883	28,80152	109,1182	26
	563,6618	11,99281	33,69391	180,2475	47
	416,6696	9,259325	27,77387	121,8096	45

; (all skls)	# junctions (all skls)	# tips (all skls)	# triple points (all skl:	# quadruple points (al	# junction '
	17	14	14	3	36
	8	10	8	0	14
	0	2	0	0	0
	11	2	8	2	30
	15	13	13	1	37
	19	11	19	0	37
	12	12	12	0	30
	5	6	4	1	14
	9	9	7	2	13
	3	5	3	0	7
	15	12	14	1	36
	23	11	23	0	43
	14	10	14	0	26
	24	20	22	2	52
	24	17	21	2	51

voxels (all s	# slab voxels (all skls)	tree length (all skls) [	average branch lengt	maximum branch len	shortest pa
	447	398,58	11,388	31,45037	113,417
	230	203,588	10,1794	30,82802	103,9392
	55	49,63615	49,63615	49,63615	49,63615
	354	319,5436	17,75242	59,37175	83,82526
	404	360,2175	10,29193	39,22902	176,9113
	396	356,7377	9,387833	36,64971	125,3182
	398	351,9843	13,03646	27,41775	153,477
	249	204,2019	15,70784	34,74193	96,86637
	233	204,2103	10,74791	43,78565	94,58852
	225	182,7042	26,1006	64,59147	154,6437
	379	332,1932	10,71591	21,66559	134,6485
	437	399,6923	9,295171	28,80152	128,8064
	287	251,001	9,653883	28,80152	109,1182
	646	566,5973	11,80411	33,69391	183,1829
	503	450,8384	8,348858	27,77387	155,9783

ath (all skl) [micron]

Datafile wa	The plugin	Plugin version: v0.1.4
Datafile wa	The plugin	Plugin version: v0.1.4
Datafile wa	The plugin	Plugin version: v0.1.4
Datafile wa	The plugin	Plugin version: v0.1.4
Datafile wa	The plugin	Plugin version: v0.1.4
Datafile wa	The plugin	Plugin version: v0.1.4
Datafile wa	The plugin	Plugin version: v0.1.4
Datafile wa	The plugin	Plugin version: v0.1.4
Datafile wa	The plugin	Plugin version: v0.1.4
Datafile wa	The plugin	Plugin version: v0.1.4
Datafile wa	The plugin	Plugin version: v0.1.4
Datafile wa	The plugin	Plugin version: v0.1.4
Datafile wa	The plugin	Plugin version: v0.1.4
Datafile wa	The plugin	Plugin version: v0.1.4
Datafile wa	The plugin	Plugin version: v0.1.4

Supplementary Table 25. MOTIQ all morphometric data for microglia in subretinally injected PBS Z2. Dataset

Number	Image	particle nr	x center [micron]	y center [micron]	x center of mass [mic	y center of
**Cell1**	MAX_C1-2(	1	205,5977	451,8746	204,2286	451,5562
**Cell2**	MAX_C1-2(	1	239,1545	237,857	239,2084	235,9762
**Cell3**	MAX_C1-2(	1	503,0727	212,8631	502,0639	212,1092
**Cell4**	MAX_C1-2(	1	119,9951	216,2672	118,5087	216,9905
**Cell5**	MAX_C1-2(	1	347,8251	520,3446	348,0891	519,3556
**Cell6**	MAX_C1-2(	1	418,9256	578,7952	418,688	577,0004
**Cell7**	MAX_C1-2(	1	175,86	350,4449	175,5611	350,6401
**Cell8**	MAX_C1-2(	1	462,5156	474,782	462,2279	473,6909
**Cell9**	MAX_C1-2(	1	146,4124	147,9909	146,7615	147,4189
**Cell10**	MAX_C1-2(	1	173,5131	451,514	173,797	449,1922
**Cell11**	MAX_C1-2(	1	364,6881	173,4562	361,9205	171,9144
**Cell12**	MAX_C1-2(	1	522,6428	527,1901	522,9138	527,0805
**Cell13**	MAX_C1-2(	1	79,70408	143,597	78,98935	144,2692
**Cell14**	MAX_C1-2(	1	378,2278	423,6904	376,665	423,5288
**Cell15**	MAX_C1-2(	1	484,418	153,7769	483,5657	153,1143

used in Figure 7.

mass [mic x span [micron]	y span [micron]	average intensity	minimum intensity	maximum
78,18338	74,03205	71,36224	14	255
106,5508	94,09681	54,84848	13	255
74,72394	65,72939	68,12141	15	255
78,87527	103,0914	96,91276	42	255
80,25904	74,72394	94,83287	39	255
106,5508	89,94548	86,19369	38	255
58,81051	75,41583	108,6994	28	255
56,04295	60,88617	120,3402	26	255
55,35106	78,18338	119,0844	27	255
94,7887	117,621	83,10662	14	240
117,621	121,7723	66,90045	12	240
80,25904	122,4642	76,34049	9	240
84,41037	98,94003	85,98311	28	255
71,95638	76,7996	102,6502	31	255
78,87527	66,42128	104,0361	32	255

intensity	standard deviation of	area [micron^2]	outline [micron]	ramification index	spanned a
	57,30973	1435,171	1845,958	13,74564	4190,144
	43,11036	1933,507	3001,411	19,25519	6458,748
	54,23336	1115,872	1390,695	11,74411	3294,478
	52,88255	1080,926	1527,689	13,10787	4287,322
	55,19515	1200,125	1721,418	14,01743	4360,564
	50,95621	1426,075	2244,486	16,76643	6154,288
	79,93239	961,7272	957,5734	8,710462	2890,926
	85,34185	819,0718	752,7745	7,419915	2745,877
	83,22296	1100,074	925,7465	7,873651	2512,267
	68,52945	2819,598	2838,126	15,07763	7073,41
	62,69829	3087,197	4169,319	21,16791	9765,193
	71,41252	2764,068	2796,613	15,00559	7018,838
	65,73904	1757,342	1811,364	12,18913	5103,521
	75,05124	1517,509	1404,533	10,17095	4257,163
	72,75447	1446,66	1237,096	9,175196	3748,295

rea (conve	spanned outline (con	spanned area center	spanned area center	polarity vector x (bina	polarity ve
	300,2795	207,8817	452,3563	2,284008	0,48166
	398,5277	238,0967	239,8951	-1,05782	2,038109
	276,7553	505,1385	213,2024	2,065784	0,339333
	361,1657	124,1877	218,4731	4,192567	2,205894
	307,1984	349,0747	522,4592	1,249628	2,114608
	390,225	417,1472	582,1714	-1,77841	3,376203
	264,3013	177,13	352,1297	1,269999	1,68486
	231,0907	464,5668	476,8359	2,051205	2,053922
	264,3013	145,9506	148,7046	-0,46176	0,713634
	422,0519	173,242	453,2867	-0,27116	1,772697
	476,0192	366,469	170,6655	1,780931	-2,79066
	402,679	522,0925	531,301	-0,55028	4,110909
	362,5495	81,28844	143,0418	1,584357	-0,55519
	293,3606	379,4982	421,5249	1,27041	-2,16548
	287,8255	486,3409	158,2503	1,922953	4,47345

ctor y (bina	polarity vector length	polarity index (binary	polarity vector x [mic	polarity vector y [mic	polarity ve
	2,334242	0,031958	3,653069	0,800064	3,739654
	2,296274	0,025322	-1,11177	3,918931	4,07358
	2,093468	0,032323	3,074604	1,093198	3,263169
	4,737466	0,064121	5,679005	1,482534	5,869327
	2,456245	0,032964	0,985599	3,103591	3,25633
	3,815951	0,043108	-1,54083	5,171028	5,39571
	2,109893	0,034777	1,568869	1,48966	2,163431
	2,902764	0,049093	2,338872	3,14502	3,919371
	0,849999	0,015029	-0,81088	1,28571	1,520058
	1,793316	0,018897	-0,55499	4,094478	4,131921
	3,310509	0,029689	4,548464	-1,24886	4,716796
	4,147575	0,043874	-0,82132	4,220561	4,299733
	1,678816	0,020826	2,299086	-1,22737	2,606189
	2,510623	0,034101	2,833255	-2,00389	3,470291
	4,86924	0,070484	2,775266	5,136028	5,837884

ctor length polarity index	ID of largest skeleton	# found skls	# branches (largest sl	# junctions
0,051199	1	5	40	23
0,044921	2	7	67	37
0,050384	2	2	36	19
0,07944	1	9	23	11
0,043702	1	6	26	13
0,060954	3	9	47	25
0,035659	2	5	18	8
0,066286	2	4	18	10
0,026876	1	3	15	8
0,043539	1	8	64	35
0,042301	2	11	130	75
0,045483	1	6	83	46
0,032331	1	8	36	18
0,047136	2	8	28	14
0,084505	1	4	26	14

s (largest sk	# tips (largest skl)	# triple points (larges	# quadruple points (la	# junction voxels (larg	# slab voxe
	9	21	2	40	416
	20	32	4	89	643
	13	17	2	42	330
	11	9	2	27	332
	12	12	1	24	386
	19	25	0	49	466
	11	7	1	20	219
	6	10	0	20	199
	6	8	0	18	222
	20	32	3	82	656
	29	68	5	152	1086
	22	40	6	90	829
	16	17	0	37	423
	13	13	1	25	332
	10	14	0	28	330

els (largest	tree length (largest sk	average branch lengt	maximum branch len	shortest path (largest	# branches
	377,0483	9,426207	28,92023	111,1039	40
	588,2583	8,779975	21,71476	186,5236	67
	305,2921	8,480335	33,93133	117,8012	35
	300,729	13,07517	31,11461	142,9445	23
	330,9955	12,7306	31,97438	104,9805	28
	420,5677	8,948249	26,60715	156,4616	47
	200,0098	11,11166	22,93066	102,2485	18
	181,0423	10,0579	26,67669	83,37079	18
	192,7552	12,85035	33,88216	101,5769	15
	610,5592	9,539988	24,7689	150,8078	64
	1004,657	7,728134	23,79042	203,3952	135
	735,4682	8,861063	27,34821	160,9108	83
	376,1309	10,44808	26,74623	118,1619	36
	291,3207	10,40431	23,33596	97,558	28
	287,4624	11,05625	22,88149	98,35492	26

s (all skls)	# junctions (all skls)	# tips (all skls)	# triple points (all skl	# quadruple points (a	# junction
	23	9	21	2	40
	37	20	32	4	89
	19	11	17	2	42
	11	11	9	2	27
	14	13	13	1	27
	25	19	25	0	49
	8	11	7	1	20
	10	6	10	0	20
	8	6	8	0	18
	35	20	32	3	82
	78	29	70	6	160
	46	22	40	6	90
	18	16	17	0	37
	14	13	13	1	25
	14	10	14	0	28

voxels (all :	# slab voxels (all skls	tree length (all skls) [	average branch lengtl	maximum branch len	shortest pa
	421	384,135	8,730342	28,92023	118,1907
	663	608,708	8,454278	21,71476	206,9733
	330	305,2921	8,480335	33,93133	117,8012
	344	314,3294	10,83894	31,11461	156,5448
	397	346,7207	10,83502	31,97438	119,3219
	488	442,352	8,191704	26,60715	178,2459
	223	205,4262	9,782201	22,93066	107,6649
	204	187,1505	8,911931	26,67669	89,47908
	224	194,8309	12,17693	33,88216	103,6526
	683	636,5932	9,361665	24,7689	176,8418
	1213	1117,976	7,71018	28,22834	311,9894
	831	740,598	8,415887	27,34821	166,0406
	440	394,5049	9,622071	26,74623	136,5359
	347	306,9967	9,029316	23,33596	113,234
	332	290,5166	10,37559	22,88149	101,4091

ath (all skl) [micron]

Datafile wa	The plugin	Plugin version: v0.1.4
Datafile wa	The plugin	Plugin version: v0.1.4
Datafile wa	The plugin	Plugin version: v0.1.4
Datafile wa	The plugin	Plugin version: v0.1.4
Datafile wa	The plugin	Plugin version: v0.1.4
Datafile wa	The plugin	Plugin version: v0.1.4
Datafile wa	The plugin	Plugin version: v0.1.4
Datafile wa	The plugin	Plugin version: v0.1.4
Datafile wa	The plugin	Plugin version: v0.1.4
Datafile wa	The plugin	Plugin version: v0.1.4
Datafile wa	The plugin	Plugin version: v0.1.4
Datafile wa	The plugin	Plugin version: v0.1.4
Datafile wa	The plugin	Plugin version: v0.1.4
Datafile wa	The plugin	Plugin version: v0.1.4
Datafile wa	The plugin	Plugin version: v0.1.4

Supplementary Table 26. MOTIQ all morphometric data for microglia in subretinally injected PBS Z3. Dataset

Number	Image	particle nr	x center [micron]	y center [micron]	x center of mass [mic	y center of
**Cell1**	MAX_C1-2(	1	328,8088	493,1351	328,2575	492,8633
**Cell2**	MAX_C1-2(	1	635,5098	347,3345	636,114	346,343
**Cell3**	MAX_C1-2(	1	335,4608	129,0615	335,0439	128,9953
**Cell4**	MAX_C1-2(	1	201,5676	328,8696	201,7543	328,982
**Cell5**	MAX_C1-2(	1	129,2504	364,2505	128,9332	365,6065
**Cell6**	MAX_C1-2(	1	510,5109	280,2746	508,3435	280,1759
**Cell7**	MAX_C1-2(	1	102,3479	356,6435	102,871	355,8914
**Cell8**	MAX_C1-2(	1	168,0647	636,282	167,3807	634,8511
**Cell9**	MAX_C1-2(	1	467,8163	332,3573	468,3026	331,8068
**Cell10**	MAX_C1-2(	1	104,2191	176,8835	103,8289	175,6345
**Cell11**	MAX_C1-2(	1	562,9143	428,9542	563,462	428,3253
**Cell12**	MAX_C1-2(	1	95,48849	176,6191	95,33782	176,593
**Cell13**	MAX_C1-2(	1	368,1279	85,0819	368,6476	84,72552
**Cell14**	MAX_C1-2(	1	550,1111	140,862	549,8415	141,2241
**Cell15**	MAX_C1-2(	1	228,1445	143,8267	228,414	143,7618

used in Figure 7.

mass [mic x span [micron]	y span [micron]	average intensity	minimum intensity	maximum
83,71848	72,64827	106,0296	37	255
79,56716	101,7076	80,77141	32	255
75,41583	71,95638	115,4053	38	255
68,49694	74,72394	87,03638	36	255
74,72394	76,10771	97,14668	35	255
98,94003	89,94548	81,95661	19	255
92,02114	98,94003	75,87207	14	255
71,95638	81,64282	111,1208	26	255
92,02114	83,71848	108,855	51	255
80,95093	103,7832	97,35916	49	255
85,10226	88,5617	121,6635	52	255
92,02114	96,17247	94,36204	43	255
82,33471	94,7887	123,3657	52	255
91,32926	71,2645	109,0109	50	255
89,94548	76,10771	122,5376	49	255

intensity	standard deviation of	area [micron^2]	outline [micron]	ramification index	spanned a
	65,98119	1180,497	1303,518	10,70236	4025,946
	53,28308	1620,91	2169,762	15,20294	6221,786
	72,55444	1241,294	1274,458	10,20431	3741,114
	55,65344	1157,998	1642,543	13,61626	3612,82
	64,08108	1360,971	1704,813	13,03609	4068,073
	63,39316	2250,413	3628,262	21,57559	5692,812
	60,94274	1837,287	3087,206	20,31759	6255,296
	72,89819	1343,259	1639,775	12,62117	3948,874
	56,29606	1610,857	2214,043	15,56154	5993,442
	49,37735	2360,516	3485,733	20,23885	6307,954
	64,86472	1389,693	1819,666	13,76979	4720,075
	55,87852	1445,224	2391,166	17,74342	5710,046
	66,32303	1455,755	1881,936	13,91412	5047,033
	59,32553	1315,493	2021,698	15,72414	4922,569
	68,66349	1223,581	1500,014	12,0969	3849,302

rea (conve	spanned outline (con	spanned area center	spanned area center	polarity vector x (bina	polarity ve
309,966	330,2522	496,0024	1,443357	2,867273	
359,7819	633,3328	348,4304	-2,17701	1,09592	
291,9769	335,0078	129,2987	-0,45298	0,237246	
282,2904	200,9297	327,368	-0,63785	-1,50161	
297,512	130,5073	358,8748	1,256973	-5,37563	
373,6197	517,7908	278,6062	7,279922	-1,66836	
377,771	101,7285	361,0044	-0,61939	4,360895	
303,0471	168,9058	638,9435	0,841089	2,661465	
347,3279	467,6359	337,1535	-0,1804	4,796186	
365,317	103,5707	182,159	-0,64841	5,275498	
343,1766	561,446	427,7865	-1,46827	-1,16772	
373,6197	97,51193	177,0689	2,023442	0,449781	
351,4793	367,4725	86,58346	-0,65546	1,501561	
322,4199	549,8324	137,862	-0,27877	-3,00001	
327,9551	227,3185	144,0096	-0,82596	0,182924	

ctor y (bina	polarity vector length	polarity index (binary	polarity vector x [mic	polarity vector y [mic	polarity ve
	3,210068	0,044836	1,994683	3,13904	3,719184
	2,437297	0,027384	-2,78114	2,087431	3,477373
	0,511352	0,007409	-0,03605	0,303425	0,305559
	1,631471	0,024055	-0,82456	-1,61399	1,812421
	5,520634	0,076708	1,574179	-6,73166	6,913267
	7,468647	0,087725	9,447373	-1,56968	9,576887
	4,404662	0,049355	-1,14246	5,11304	5,239121
	2,791205	0,039364	1,525105	4,092358	4,367303
	4,799578	0,054943	-0,66663	5,346677	5,388076
	5,315196	0,059309	-0,25813	6,524503	6,529607
	1,875998	0,024199	-2,016	-0,53879	2,086754
	2,072829	0,02431	2,174119	0,475918	2,225599
	1,638388	0,020438	-1,17514	1,857937	2,198383
	3,012929	0,038057	-0,00916	-3,36214	3,362149
	0,845976	0,012084	-1,09547	0,247859	1,123161

ctor length polarity index	ID of largest skeleton	# found skls	# branches (largest sl	# junctions
0,051947	1	3	22	10
0,03907	1	6	46	24
0,004427	1	3	35	18
0,026723	1	2	27	14
0,096058	1	5	43	24
0,112488	1	2	82	48
0,058706	3	10	36	20
0,061592	1	3	31	17
0,061679	1	10	38	21
0,07286	1	4	65	36
0,026918	1	3	34	19
0,026102	3	9	45	24
0,027424	1	3	30	15
0,042468	2	4	53	29
0,016043	1	5	22	11

s (largest sl	# tips (largest skl)	# triple points (larges	# quadruple points (la	# junction voxels (larg	# slab voxe
	13	9	1	19	307
	18	22	2	56	477
	12	16	0	44	337
	12	14	0	30	364
	11	21	3	53	416
	14	43	4	92	760
	12	20	0	38	490
	9	15	2	31	358
	12	20	1	44	463
	19	33	3	76	725
	11	19	0	37	426
	15	21	3	43	503
	13	14	0	32	419
	18	28	1	58	448
	10	10	1	14	328

els (largest	tree length (largest sk	average branch lengt	maximum branch len	shortest path (largest	# branches
	265,1253	12,05115	24,43314	103,4644	22
	434,233	9,439849	31,28249	147,1313	46
	305,4652	8,727577	30,03109	102,625	35
	320,3457	11,86466	36,29359	109,8101	27
	374,7268	8,714576	25,00632	135,2845	43
	695,9158	8,486778	30,66014	165,9534	82
	426,8151	11,85597	38,08266	143,7906	40
	326,2742	10,52497	24,65019	119,5745	31
	402,9263	10,60332	35,21677	141,7844	38
	653,3376	10,05135	26,60715	144,0031	65
	377,0195	11,08881	43,95353	142,0015	34
	460,1567	10,22571	31,21295	126,7104	45
	366,6977	12,22326	32,16262	166,7167	30
	408,891	7,714924	19,82734	131,9997	52
	281,4525	12,79329	37,22289	114,7516	22

s (all skls)	# junctions (all skls)	# tips (all skls)	# triple points (all skl	# quadruple points (a	# junction
	10	13	9	1	19
	24	18	22	2	56
	18	12	16	0	44
	14	12	14	0	30
	24	11	21	3	53
	48	14	43	4	92
	22	14	22	0	40
	17	9	15	2	31
	21	12	20	1	44
	36	19	33	3	76
	19	11	19	0	37
	24	15	21	3	43
	15	13	14	0	32
	29	16	28	1	58
	11	10	10	1	14

voxels (all s	# slab voxels (all skls	tree length (all skls) [	average branch lengt	maximum branch len	shortest pa
	309	267,8929	11,1622	24,43314	106,2319
	482	442,2983	8,672515	31,28249	155,1965
	337	305,4652	8,727577	30,03109	102,625
	373	328,6976	11,7392	36,29359	118,1619
	423	383,1973	8,153134	25,00632	143,7551
	760	695,9158	8,486778	30,66014	165,9534
	568	497,8506	10,59257	38,08266	210,2203
	359	328,6365	9,958681	24,65019	121,9367
	496	435,1873	9,670828	35,21677	174,0454
	728	657,7755	9,673169	26,60715	148,441
	436	386,4685	10,73524	43,95353	151,4505
	548	503,705	9,503867	31,21295	170,2586
	419	368,0815	11,50255	32,16262	168,1004
	453	414,3074	7,532861	19,82734	137,4161
	333	287,8473	11,51389	37,22289	121,1465

ath (all skl) [micron]

Datafile wa	The plugin	Plugin version: v0.1.4
Datafile wa	The plugin	Plugin version: v0.1.4
Datafile wa	The plugin	Plugin version: v0.1.4
Datafile wa	The plugin	Plugin version: v0.1.4
Datafile wa	The plugin	Plugin version: v0.1.4
Datafile wa	The plugin	Plugin version: v0.1.4
Datafile wa	The plugin	Plugin version: v0.1.4
Datafile wa	The plugin	Plugin version: v0.1.4
Datafile wa	The plugin	Plugin version: v0.1.4
Datafile wa	The plugin	Plugin version: v0.1.4
Datafile wa	The plugin	Plugin version: v0.1.4
Datafile wa	The plugin	Plugin version: v0.1.4
Datafile wa	The plugin	Plugin version: v0.1.4
Datafile wa	The plugin	Plugin version: v0.1.4
Datafile wa	The plugin	Plugin version: v0.1.4

Supplementary Table 27. The area of microglia (micron^2) in the different zones. Dataset used in Figure 7A.

Area						
Blood-Z1	Blood-Z2	Blood-Z3	PBS-Z1	PBS-Z2	PBS-Z3	Z4
1368,63	1718,57	1783,19	1571,12	1435,17	1180,5	1499,8
727,638	2076,16	1589,79	749,659	1933,51	1620,91	2264,77
1510,81	1410,76	1828,19	2984,27	1115,87	1241,29	2798,54
1094,81	1109,65	2175,73	2875,13	1080,93	1158	1210,66
1106,3	326,48	2453,86	1308,31	1200,12	1360,97	3147,51
453,338	696,043	1833,46	1368,15	1426,08	2250,41	2801,41
1344,69	1672,13	1798,99	1555,33	961,727	1837,29	1821,01
833,433	1447,62	1731,49	1156,56	819,072	1343,26	2681,73
1630,96	1780,32	1687,93	772,637	1100,07	1610,86	1784,15
542,378	2062,28	1231,24	850,188	2819,6	2360,52	2436,15
858,326	2015,37	2398,81	1488,31	3087,2	1389,69	3393,57
1370,55	1419,85	1268,1	1660,16	2764,07	1445,22	1653,94
1229,8	826,252	1747,29	1158	1757,34	1455,76	3299,74
1163,26	1245,12	1982,81	2386,85	1517,51	1315,49	1387,3
964,121	1671,17	2238,45	1938,29	1446,66	1223,58	1481,13

Supplementary Table 28. The outline of microglia (micron) in the different zones. Dataset used in Figure 7B.

Outline						
Blood-Z1	Blood-Z2	Blood-Z3	PBS-Z1	PBS-Z2	PBS-Z3	Z4
1675,75	1923,45	2310,91	2184,98	1845,96	1303,52	2032,77
332,106	1887,47	1837,66	1140,23	3001,41	2169,76	3309,99
1025,38	1165,14	2220,96	3891,18	1390,7	1274,46	3887,03
734,785	830,266	3013,87	4149,95	1527,69	1642,54	1721,42
934,049	261,534	2803,53	1819,67	1721,42	1704,81	4554,01
168,821	568,732	2245,87	1857,03	2244,49	3628,26	3900,87
756,926	1299,37	2204,36	1653,61	957,573	3087,21	2360,72
283,674	1224,64	1880,55	1003,24	752,774	1639,78	3524,48
1574,74	1681,29	2333,05	604,71	925,747	2214,04	2522,62
243,545	1906,84	1279,99	723,715	2838,13	3485,73	3491,27
444,192	1938,67	2824,29	1614,87	4169,32	1819,67	4206,68
1466,8	1356,1	1475,11	1815,51	2796,61	2391,17	2644,4
1345,03	799,823	1962,2	1154,07	1811,36	1881,94	4898,57
1399	1368,56	2352,42	2378,71	1404,53	2021,7	2097,81
1255,09	1675,75	2709,43	1953,89	1237,1	1500,01	2136,55

Supplementary Table 29. The ramification index of microglia in the different zones. Dataset used in Figure 7E.

Ramification Index						
Blood-Z1	Blood-Z2	Blood-Z3	PBS-Z1	PBS-Z2	PBS-Z3	Z4
12,778	13,0886	15,4376	15,5503	13,7456	10,7024	14,807
3,47308	11,6854	13,0014	11,7478	19,2552	15,2029	19,6205
7,44175	8,75079	14,653	20,0936	11,7441	10,2043	20,7275
6,2645	7,03104	18,227	21,8328	13,1079	13,6163	13,9563
7,92189	4,08314	15,9652	14,1916	14,0174	13,0361	22,8984
2,23671	6,08114	14,796	14,1627	16,7664	21,5756	20,7906
5,82286	8,96378	14,661	11,8282	8,71046	20,3176	15,6057
2,77191	9,07982	12,7488	8,32175	7,41992	12,6212	19,1992
10,9997	11,2406	16,0192	6,13698	7,87365	15,5615	16,8474
2,95001	11,845	10,2904	7,00173	15,0776	20,2388	19,9538
4,27701	12,1821	16,2669	11,8083	21,1679	13,7698	20,3707
11,1769	10,1523	11,6853	12,5695	15,0056	17,7434	18,3426
10,8196	7,84933	13,242	9,56694	12,1891	13,9141	24,056
11,5711	10,9409	14,9028	13,7349	10,1709	15,7241	15,8882
11,4026	11,5636	16,1548	12,5195	9,1752	12,0969	15,6607

Supplementary Table 31. The spanned area of microglia (micron^2) in the different zones. Dataset used in Figure 7C.

Spanned Area						
Blood-Z1	Blood-Z2	Blood-Z3	PBS-Z1	PBS-Z2	PBS-Z3	Z4
4373,49	5846,96	5911,58	4824,43	4190,14	4025,95	4638,22
1075,66	4744,01	4799,54	3154,7	6458,75	6221,79	7550,68
3139,86	3350,01	6761,29	4094,4	3294,48	3741,11	10441,1
1795,64	2034,52	9174,94	4317	4287,32	3612,82	3927,81
3009,65	522,751	7105,48	5271,07	4360,56	4068,07	10490,4
488,762	1694,15	6783,31	4108,28	6154,29	5692,81	8534,43
2316,47	4730,13	6478,85	6161,47	2890,93	6255,3	4481,2
1101,99	3810,53	5471,17	3164,75	2745,88	3948,87	7261,06
4071,42	4114,03	6004,45	1834,89	2512,27	5993,44	5887,17
873,166	5683,24	4000,1	2585,03	7073,41	6307,95	8439,65
1281,51	7038,94	7366,86	4632,47	9765,19	4720,07	8689,05
4648,27	4017,81	3951,27	4520,45	7018,84	5710,05	5947,01
3209,27	1819,57	5241,87	3067,57	5103,52	5047,03	10791,5
3404,58	3588,88	6829,27	7570,31	4257,16	4922,57	4346,2
3040,28	4723,43	7382,18	6010,68	3748,29	3849,3	4578,86

Supplementary Table 32. The spanned outline of microglia (micron) in the different zones. Dataset used in Figure 7D.

Spanned Outline						
**Blood-Z1**	**Blood-Z2**	**Blood-Z3**	**PBS-Z1**	**PBS-Z2**	**PBS-Z3**	**Z4**
321,036	361,166	375,003	308,582	300,28	309,966	340,409
149,448	314,117	340,409	278,139	398,528	359,782	430,355
261,534	272,604	408,214	271,22	276,755	291,977	506,462
214,485	211,718	455,263	312,734	361,166	282,29	283,674
253,231	106,551	404,063	361,166	307,198	297,512	513,381
109,318	197,88	405,447	339,025	390,225	373,62	446,96
235,242	368,085	398,528	362,549	264,301	377,771	309,966
159,134	314,117	351,479	273,988	231,091	303,047	431,738
285,058	305,815	424,819	218,637	264,301	347,328	359,782
128,691	343,177	301,663	316,885	422,052	365,317	415,133
184,042	441,425	410,982	333,49	476,019	343,177	456,646
351,479	307,198	340,409	322,42	402,679	373,62	370,852
286,442	210,334	373,62	275,372	362,549	351,479	507,846
251,847	308,582	392,993	405,447	293,361	322,42	300,28
251,847	352,863	413,749	366,701	287,826	327,955	318,269

Supplementary Table 33. ANOVA results for microglia area morphometric data in the different zones. Results used in Figure 7A.

Cases	Sum of Squares	df	Mean Square	F	p
Zone	$1.171\times10^{+7}$	6	$1.952\times10^{+6}$	6.195	< .001
Residuals	$3.088\times10^{+7}$	98	315055.386		

Note. Type III Sum of Squares

Supplementary Table 34. Tukey's post hoc comparasion results for microglia area morphometric data in the different zones. Results used in Figure 7A.

		Mean Difference	95% CI for Mean Difference Lower	Upper	SE	df	t	p_{tukey}
BZ1	BZ2	−351.915	−968.932	265.101	204.957	98	−1.717	0.606
	BZ3	−770.020	−1387.037	−153.004	204.957	98	−3.757	0.005**
	PZ1	−508.262	−1125.278	108.755	204.957	98	−2.480	0.178
	PZ2	−551.058	−1168.075	65.958	204.957	98	−2.689	0.112
	PZ3	−439.647	−1056.663	177.370	204.957	98	−2.145	0.335
	Z4	−1164.157	−1781.174	−547.141	204.957	98	−5.680	< .001***
BZ2	BZ3	−418.105	−1035.121	198.912	204.957	98	−2.040	0.397
	PZ1	−156.346	−773.363	460.670	204.957	98	−0.763	0.988
	PZ2	−199.143	−816.160	417.873	204.957	98	−0.972	0.959
	PZ3	−87.731	−704.748	529.285	204.957	98	−0.428	1.000
	Z4	−812.242	−1429.259	−195.226	204.957	98	−3.963	0.003**
BZ3	PZ1	261.758	−355.258	878.775	204.957	98	1.277	0.861
	PZ2	218.962	−398.055	835.978	204.957	98	1.068	0.936
	PZ3	330.373	−286.643	947.390	204.957	98	1.612	0.675
	Z4	−394.137	−1011.154	222.879	204.957	98	−1.923	0.470
PZ1	PZ2	−42.797	−659.813	574.220	204.957	98	−0.209	1.000
	PZ3	68.615	−548.401	685.632	204.957	98	0.335	1.000
	Z4	−655.896	−1272.912	−38.879	204.957	98	−3.200	0.030*
PZ2	PZ3	111.412	−505.605	728.428	204.957	98	0.544	0.998
	Z4	−613.099	−1230.116	3.917	204.957	98	−2.991	0.053
PZ3	Z4	−724.511	−1341.527	−107.494	204.957	98	−3.535	0.011*

* p < .05, ** p < .01, *** p < .001

Note. P-value and confidence intervals adjusted for comparing a family of 7 estimates (confidence intervals corrected using the tukey method).

Supplementary Table 35. ANOVA results for microglia outline morphometric data in the different zones. Results used in Figure 7B.

Cases	Sum of Squares	df	Mean Square	F	p
Zone	$4.548 \times 10^{+7}$	6	$7.580 \times 10^{+6}$	12.373	< .001
Residuals	$6.004 \times 10^{+7}$	98	612615.603		

Note. Type III Sum of Squares

Supplementary Table 36. Tukey's post hoc comparasion results for microglia outline morphometric data in the different zones. Results used in Figure 7B.

			95% CI for Mean Difference					
		Mean Difference	Lower	Upper	SE	df	t	p_{tukey}
BZ1	BZ2	−416.517	−1276.911	443.877	285.801	98	−1.457	0.769
	BZ3	−1320.953	−2181.347	−460.559	285.801	98	−4.622	< .001***
	PZ1	−953.699	−1814.093	−93.305	285.801	98	−3.337	0.020*
	PZ2	−998.994	−1859.388	−138.600	285.801	98	−3.495	0.012*
	PZ3	−1208.314	−2068.708	−347.920	285.801	98	−4.228	0.001**
	Z4	−2243.286	−3103.680	−1382.892	285.801	98	−7.849	< .001***
BZ2	BZ3	−904.436	−1764.830	−44.042	285.801	98	−3.165	0.033*
	PZ1	−537.182	−1397.576	323.212	285.801	98	−1.880	0.499
	PZ2	−582.478	−1442.872	277.916	285.801	98	−2.038	0.398
	PZ3	−791.797	−1652.191	68.597	285.801	98	−2.770	0.092
	Z4	−1826.770	−2687.164	−966.376	285.801	98	−6.392	< .001***
BZ3	PZ1	367.254	−493.140	1227.648	285.801	98	1.285	0.857
	PZ2	321.959	−538.435	1182.353	285.801	98	1.127	0.918
	PZ3	112.639	−747.755	973.033	285.801	98	0.394	1.000
	Z4	−922.333	−1782.727	−61.939	285.801	98	−3.227	0.027*
PZ1	PZ2	−45.296	−905.690	815.098	285.801	98	−0.158	1.000
	PZ3	−254.615	−1115.009	605.779	285.801	98	−0.891	0.973
	Z4	−1289.588	−2149.982	−429.194	285.801	98	−4.512	< .001***
PZ2	PZ3	−209.319	−1069.713	651.075	285.801	98	−0.732	0.990
	Z4	−1244.292	−2104.686	−383.898	285.801	98	−4.354	< .001***
PZ3	Z4	−1034.973	−1895.367	−174.579	285.801	98	−3.621	0.008**

* p < .05, ** p < .01, *** p < .001

Note. P-value and confidence intervals adjusted for comparing a family of 7 estimates (confidence intervals corrected using the tukey method).

Supplementary Table 37. ANOVA results for microglia ramification index morphometric data in the different zones. Results used in Figure 7E.

Cases	Sum of Squares	df	Mean Square	F	p
Zone	1199.253	6	199.875	17.251	< .001
Residuals	1135.490	98	11.587		

Note. Type III Sum of Squares

Supplementary Table 38. Tukey's post hoc comparasion results for microglia ramification index morphometric data in the different zones. Results used in Figure 7E.

		Mean Difference	95% CI for Mean Difference Lower	Upper	SE	df	t	p_{tukey}
BZ1	BZ2	−2.175	−5.917	1.566	1.243	98	−1.750	0.584
	BZ3	−7.076	−10.818	−3.334	1.243	98	−5.693	< .001***
	PZ1	−5.277	−9.019	−1.535	1.243	98	−4.246	< .001***
	PZ2	−5.568	−9.310	−1.826	1.243	98	−4.480	< .001***
	PZ3	−7.628	−11.370	−3.886	1.243	98	−6.137	< .001***
	Z4	−11.121	−14.863	−7.379	1.243	98	−8.948	< .001***
BZ2	BZ3	−4.901	−8.643	−1.159	1.243	98	−3.943	0.003**
	PZ1	−3.102	−6.844	0.640	1.243	98	−2.496	0.172
	PZ2	−3.393	−7.134	0.349	1.243	98	−2.730	0.102
	PZ3	−5.452	−9.194	−1.711	1.243	98	−4.387	< .001***
	Z4	−8.946	−12.688	−5.204	1.243	98	−7.197	< .001***
BZ3	PZ1	1.799	−1.943	5.541	1.243	98	1.447	0.775
	PZ2	1.508	−2.234	5.250	1.243	98	1.213	0.887
	PZ3	−0.552	−4.293	3.190	1.243	98	−0.444	0.999
	Z4	−4.045	−7.787	−0.303	1.243	98	−3.254	0.025*
PZ1	PZ2	−0.291	−4.033	3.451	1.243	98	−0.234	1.000
	PZ3	−2.351	−6.092	1.391	1.243	98	−1.891	0.491
	Z4	−5.844	−9.586	−2.102	1.243	98	−4.702	< .001***
PZ2	PZ3	−2.060	−5.802	1.682	1.243	98	−1.657	0.646
	Z4	−5.553	−9.295	−1.811	1.243	98	−4.468	< .001***
PZ3	Z4	−3.493	−7.235	0.248	1.243	98	−2.811	0.084

* p < .05, ** p < .01, *** p < .001

Note. P-value and confidence intervals adjusted for comparing a family of 7 estimates (confidence intervals corrected using the tukey method).

Supplementary Table 39. ANOVA results for microglia spanned area morphometric data in the different zones. Results used in Figure 7C.

Cases	Sum of Squares	df	Mean Square	F	p
Zone	$2.020\times10^{+8}$	6	$3.367\times10^{+7}$	11.721	< .001
Residuals	$2.815\times10^{+8}$	98	$2.873\times10^{+6}$		

Note. Type III Sum of Squares

Supplementary Table 40. Tukey's post hoc comparasion results for microglia spanned area morphometric data in the different zones. Results used in Figure 7C.

		Mean Difference	95% CI for Mean Difference Lower	Upper	SE	df	t	p_{tukey}
BZ1	BZ2	−1325.929	−3189.025	537.166	618.873	98	−2.142	0.337
	BZ3	−3695.477	−5558.573	−1832.381	618.873	98	−5.971	< .001***
	PZ1	−1832.500	−3695.595	30.596	618.873	98	−2.961	0.057
	PZ2	−2402.068	−4265.164	−538.972	618.873	98	−3.881	0.003**
	PZ3	−2419.142	−4282.238	−556.046	618.873	98	−3.909	0.003**
	Z4	−4544.963	−6408.059	−2681.867	618.873	98	−7.344	< .001***
BZ2	BZ3	−2369.548	−4232.644	−506.452	618.873	98	−3.829	0.004**
	PZ1	−506.570	−2369.666	1356.526	618.873	98	−0.819	0.983
	PZ2	−1076.139	−2939.235	786.957	618.873	98	−1.739	0.592
	PZ3	−1093.213	−2956.309	769.883	618.873	98	−1.766	0.574
	Z4	−3219.034	−5082.129	−1355.938	618.873	98	−5.201	< .001***
BZ3	PZ1	1862.977	−0.118	3726.073	618.873	98	3.010	0.050
	PZ2	1293.409	−569.687	3156.505	618.873	98	2.090	0.367
	PZ3	1276.335	−586.761	3139.431	618.873	98	2.062	0.383
	Z4	−849.486	−2712.582	1013.610	618.873	98	−1.373	0.815
PZ1	PZ2	−569.568	−2432.664	1293.527	618.873	98	−0.920	0.968
	PZ3	−586.642	−2449.738	1276.453	618.873	98	−0.948	0.964
	Z4	−2712.463	−4575.559	−849.368	618.873	98	−4.383	< .001***
PZ2	PZ3	−17.074	−1880.170	1846.022	618.873	98	−0.028	1.000
	Z4	−2142.895	−4005.991	−279.799	618.873	98	−3.463	0.014*
PZ3	Z4	−2125.821	−3988.917	−262.725	618.873	98	−3.435	0.015*

* p < .05, ** p < .01, *** p < .001

Note. P-value and confidence intervals adjusted for comparing a family of 7 estimates (confidence intervals corrected using the tukey method).

Supplementary Table 41. ANOVA results for microglia spanned outline morphometric data in the different zones. Results used in Figure 7D.

Cases	Sum of Squares	df	Mean Square	F	p
Zone	293944.144	6	48990.691	12.155	< .001
Residuals	394988.123	98	4030.491		

Note. Type III Sum of Squares

Supplementary Table 42. Tukey's post hoc comparasion results for microglia spanned outline morphometric data in the different zones. Results used in Figure 7D.

			95% CI for Mean Difference					
		Mean Difference	Lower	Upper	SE	df	t	p_{tukey}
BZ1	BZ2	−64.853	−134.641	4.935	23.182	98	−2.798	0.086
	BZ3	−156.920	−226.709	−87.132	23.182	98	−6.769	< .001***
	PZ1	−86.901	−156.689	−17.113	23.182	98	−3.749	0.005**
	PZ2	−106.366	−176.155	−36.578	23.182	98	−4.588	< .001***
	PZ3	−105.628	−175.417	−35.840	23.182	98	−4.557	< .001***
	Z4	−169.928	−239.716	−100.140	23.182	98	−7.330	< .001***
BZ2	BZ3	−92.067	−161.856	−22.279	23.182	98	−3.972	0.003**
	PZ1	−22.048	−91.836	47.740	23.182	98	−0.951	0.963
	PZ2	−41.513	−111.302	28.275	23.182	98	−1.791	0.557
	PZ3	−40.775	−110.564	29.013	23.182	98	−1.759	0.579
	Z4	−105.075	−174.863	−35.287	23.182	98	−4.533	< .001***
BZ3	PZ1	70.019	0.231	139.807	23.182	98	3.020	0.049*
	PZ2	50.554	−19.234	120.342	23.182	98	2.181	0.315
	PZ3	51.292	−18.496	121.080	23.182	98	2.213	0.298
	Z4	−13.008	−82.796	56.781	23.182	98	−0.561	0.998
PZ1	PZ2	−19.465	−89.253	50.323	23.182	98	−0.840	0.980
	PZ3	−18.727	−88.515	51.061	23.182	98	−0.808	0.984
	Z4	−83.027	−152.815	−13.238	23.182	98	−3.582	0.009**
PZ2	PZ3	0.738	−69.050	70.526	23.182	98	0.032	1.000
	Z4	−63.561	−133.350	6.227	23.182	98	−2.742	0.099
PZ3	Z4	−64.299	−134.088	5.489	23.182	98	−2.774	0.092

* p < .05, ** p < .01, *** p < .001

Note. P-value and confidence intervals adjusted for comparing a family of 7 estimates (confidence intervals corrected using the tukey method).
